# Optimizing aspirin dose for colorectal cancer patients through deep phenotyping using novel biomarkers of drug action

**DOI:** 10.3389/fphar.2024.1362217

**Published:** 2024-02-29

**Authors:** Paola Patrignani, Stefania Tacconelli, Annalisa Contursi, Elena Piazuelo, Annalisa Bruno, Stefania Nobili, Matteo Mazzei, Cristina Milillo, Ulrika Hofling, Gonzalo Hijos-Mallada, Carlos Sostres, Angel Lanas

**Affiliations:** ^1^ Systems Pharmacology and Translational Therapeutics Laboratory, at the Center for Advanced Studies and Technology (CAST), “G. d’Annunzio” University, Chieti, Italy; ^2^ Department of Neuroscience, Imaging and Clinical Science, “G. d’Annunzio” University Medical School, Chieti, Italy; ^3^ Instituto Aragonés de Ciencias de la Salud (IACS), Zaragoza, Spain; ^4^ Department of Psychological Sciences, Health, and Territory, “G. d’Annunzio” University, Chieti, Italy; ^5^ University Hospital LB, Aragon Health Research Institute (IISAragon), CIBERehd, University of Zaragoza, Zaragoza, Spain

**Keywords:** colorectal cancer, biomarker-based aspirin dose optimization, thromboxane A2, prostaglandin E2, cyclooxygenases, platelets, acetylation of COX-1

## Abstract

**Background:** Low-dose aspirin’s mechanism of action for preventing colorectal cancer (CRC) is still debated, and the optimal dose remains uncertain. We aimed to optimize the aspirin dose for cancer prevention in CRC patients through deep phenotyping using innovative biomarkers for aspirin’s action.

**Methods:** We conducted a Phase II, open-label clinical trial in 34 CRC patients of both sexes randomized to receive enteric-coated aspirin 100 mg/d, 100 mg/BID, or 300 mg/d for 3 ± 1 weeks. Biomarkers were evaluated in blood, urine, and colorectal biopsies at baseline and after dosing with aspirin. Novel biomarkers of aspirin action were assessed in platelets and colorectal tissues using liquid chromatography-mass spectrometry to quantify the extent of cyclooxygenase (COX)-1 and COX-2 acetylation at Serine 529 and Serine 516, respectively.

**Results:** All aspirin doses caused comparable % acetylation of platelet COX-1 at Serine 529 associated with similar profound inhibition of platelet-dependent thromboxane (TX)A_2_ generation *ex vivo* (serum TXB_2_) and *in vivo* (urinary TXM). TXB_2_ was significantly reduced in CRC tissue by aspirin 300 mg/d and 100 mg/BID, associated with comparable % acetylation of COX-1. Differently, 100 mg/day showed a lower % acetylation of COX-1 in CRC tissue and no significant reduction of TXB_2_. Prostaglandin (PG)E_2_ biosynthesis in colorectal tumors and *in vivo* (urinary PGEM) remained unaffected by any dose of aspirin associated with the variable and low extent of COX-2 acetylation at Serine 516 in tumor tissue. Increased expression of tumor-promoting genes like *VIM* (vimentin) and *TWIST1* (Twist Family BHLH Transcription Factor 1) *vs*. baseline was detected with 100 mg/d of aspirin but not with the other two higher doses.

**Conclusion:** In CRC patients, aspirin 300 mg/d or 100 mg/BID had comparable antiplatelet effects to aspirin 100 mg/d, indicating similar inhibition of the platelet’s contribution to cancer. However, aspirin 300 mg/d and 100 mg/BID can have additional anticancer effects by inhibiting cancerous tissue’s TXA_2_ biosynthesis associated with a restraining impact on tumor-promoting gene expression. EUDRACT number: 2018-002101-65.

**Clinical Trial Registration:** ClinicalTrials.gov, identifier NCT03957902.

## 1 Introduction

As a prevention therapy, low-dose aspirin (75–100 mg/d) has been recommended for cardiovascular disease (CVD) for a long time (Patrono, 1994), and it has also been considered for colorectal cancer (CRC) more recently (Patrignani and Patrono, 2016). Ongoing discussion exists regarding how low-dose aspirin works as an anticancer agent (Patrono, 1994; Patrignani and Patrono, 2018). As a result, it is uncertain whether higher doses of aspirin provide greater efficacy in preventing cancer than low doses (Patrignani and Patrono, 2016).

Aspirin, also known as acetylsalicylic acid or ASA, is a nonsteroidal antiinflammatory drug (NSAID) that works by acetylating cyclooxygenase (COX)-1 and COX-2 enzymes, at Serine 529 and Serine 516, respectively, of the COX active site (Roth and Majerus, 1975; Lecomte et al., 1994). This process leads to the irreversible inactivation of COX-isozyme activity and inhibition of prostanoid biosynthesis (Patrono, 1994; Bruno et al., 2023). Due to the short pharmacological half-life of the drug (approximately 20 min) (Brunton et al., 2017), the duration of this effect depends on the time necessary for the acetylated proteins to be newly synthesized by the cells. When taken at low doses, such as 100 mg daily, aspirin mainly targets platelet COX-1 (Patrignani et al., 1982; Patrignani et al., 2014). The drug circulates at low concentrations (about 4–10 μM), but an important degree of inhibition of platelet COX-1 occurs in the presystemic circulation (Pedersen and FitzGerald, 1984; Patrignani et al., 2014). This phenomenon and the irreversible nature of COX-1 inhibition in the anucleated platelet, which has limited *de novo* protein synthesis (Evangelista et al., 2006), leads to a maximum inhibition of platelet COX-1 activity by low-dose aspirin. This effect persists throughout the dosing interval of 24 h (Patrignani et al., 1982; Patrignani et al., 2014). The inhibition of COX-1/thromboxane (TX)A_2_-dependent platelet function is the most plausible mechanism for the secondary prevention of cardiovascular disease by low-dose aspirin (Patrono, 1994).

The benefit of low-dose aspirin in cancer prevention has led to the proposal that the drug can limit the development of colorectal tumors by affecting platelet-dependent signaling pathways triggered during the early stages of the development of colorectal adenomatous lesions (Patrignani and Patrono, 2016; Patrignani and Patrono, 2018). Our recent findings support this hypothesis. *Apc* ^*Min*/+^ mice (an animal model bearing multiple intestinal neoplasia) with the specific deletion of *Ptgs1* (protein name COX-1) in the megakaryocytes/platelets (mimicking the pharmacodynamics of low-dose aspirin in humans) were associated with reduced number and size of tumors of the small intestine (Bruno et al., 2022). One of the first events in developing intestinal adenomatous lesions is the induction of COX-2 in myofibroblasts, leading to enhanced biosynthesis of prostaglandin (PG) E_2_, which is a crucial pathway in cancer as it prevents apoptosis while promoting migration, proliferation, angiogenesis, and immune evasion (Wang and Dubois, 2010; Wang and Dubois, 2014). Extravasated platelets are detected in conjunction with myofibroblasts in the inflamed colon (Sacco et al., 2019) and in intestine tissue sections of *Apc* ^*Min/+*^ mice (Bruno et al., 2022). The release of platelet TXA_2_ activates myofibroblast TXA_2_ receptors (TP) involved in COX-2 expression, enhances proliferative and migratory abilities, and expression of mesenchymal markers, such as vimentin, in myofibroblasts (Sacco et al., 2019; Bruno et al., 2022). Low-dose aspirin can indirectly prevent the upregulation of COX-2-dependent signaling pathways that promote cancer development by affecting platelet function (Patrignani and Patrono, 2016; Patrignani and Patrono, 2018).

However, it is still unknown whether higher doses of aspirin can improve antitumor effects by affecting prostanoid biosynthesis in extraplatelet cellular sources, including colorectal adenomas and carcinomas, by directly inhibiting COX-1 and COX-2 activity. A detailed understanding of the mechanisms of action of low and higher aspirin doses will allow for improving aspirin chemoprevention by selecting the appropriate dose and considering the individuals’ efficacy and safety. To clarify the aspirin mechanism of action, we carried out a biomarker program during the last years to develop novel direct biomarkers of aspirin action involving the quantitative assessment of the extent of acetylation of COX-1 and COX-2 at Serine 529 and Serine 516, respectively, by liquid chromatography-mass spectrometry (LC-MS/MS) (Patrignani et al., 2014; Patrignani et al., 2017; Tacconelli et al., 2020). These innovative assays permit the identification of the cellular and biochemical targets associated with aspirin administration, i.e., the platelet *vs.* the colorectum and the COX-1 *vs*. the COX-2 pathway.

**FIGURE 1:**
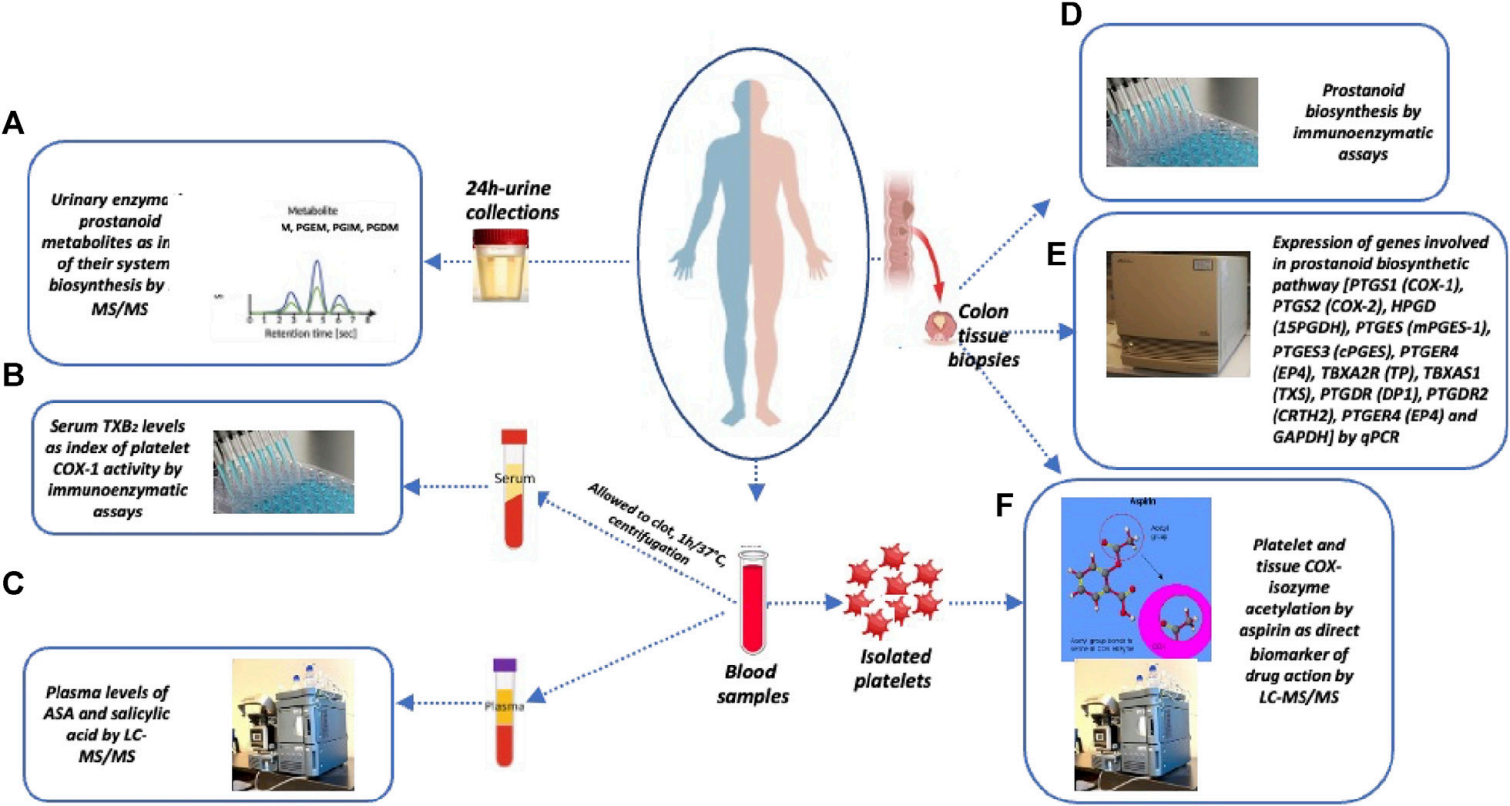
Sample collection and biomarker evaluation. At baseline and after the last Aspirin dose, the following samples were collected: i) urine samples (overnight or 24 h collections), ii) blood samples, and iii) colon tissue biopsies for the assessment of the following biomarkers: **(A)** systemic biosynthesis of TXA_2_, PGI_2_, PGE_2_, and PGD_2_ by assessing their urinary primary enzymatic metabolites, i.e., 11-dehydro-TXB_2_ and 2,3-dinor-TXB_2_ (TXM), 2,3-dinor-6-keto-PGF_1α_ (PGIM), 7-hydroxy-5,11-diketotetranorprostane-1,16-dioic acid (PGEM), and 11,15-dioxo-9α-hydroxy-2,3,4,5-tetranorprostan-1,20-dioic acid (PGDM), by liquid chromatography-mass spectrometry (LC-MS/MS); **(B)** platelet COX-1 activity *ex vivo* by evaluating the production of TXB_2_ in serum by allowing whole blood to clot for 1 h at 37°C; **(C)** plasma levels of acetylsalicylic acid (ASA) and salicylic acid (SA) by LC-MS/MS; **(D)** PGE_2_ and TXB_2_ levels in colorectal biopsies of normal and tumor tissue by specific immunoassays; **(E)** the expression of genes involved in prostanoid biosynthetic pathways and epithelial-mesenchymal transition (EMT) marker genes and GAPDH by qPCR; **(F)** % acetylation of washed platelet COX-1 at Serine529 and % acetylation of COX-1 at Serine529 and COX-2 at Serine516 in the biopsies of colorectal tumor and normal tissue by LC-MS/MS.

Here, we compared the effects of two doses of aspirin (100 mg/d and 300 mg/d) given for ∼3 weeks to CRC patients on acetylation of COX-isozymes at Serine 529 and 516 in platelets and colorectal cancer tissue. We chose these two doses because they are currently being used in clinical trials to evaluate aspirin’s efficacy against cancer (Coyle et al., 2016; CAPP3). We also compared the impact of the two doses of aspirin on prostanoid biosynthesis both *ex vivo* and *in vivo*. In colorectal cancer tissue, the inhibition of prostanoid biosynthesis and the changes in the expression of enzymes and receptors involved in prostanoid pathways and epithelial-mesenchymal transition (EMT) markers were evaluated. EMT represents a crucial event during cancer progression and dissemination (Gröger et al., 2012). Additionally, we studied the effects of taking 100 mg of aspirin twice daily (BID) on the same biomarkers to verify whether more frequent dosing leads to enhanced and longer-lasting effects. Our biomarker strategy is illustrated in Figure 1. We found that all aspirin doses had comparable antiplatelet effects. However, aspirin 300 mg/d and 100 mg/BID, but not 100 mg/d, significantly inhibited cancerous tissue’s TXA_2_ biosynthesis associated with a restraining impact on tumor-promoting gene expression.

## 2 Materials and methods

### 2.1 Patients and treatments

We conducted a Phase II, open-label clinical trial in 42 patients with newly diagnosed CRC randomized to 3 groups of patients receiving 100 mg/d (*n* = 14), 300 mg/d (*n* = 14), or 100 mg/BID (*n* = 14) of enteric-coated (EC)-aspirin (Adiro^®^) for 3 ± 1 weeks, before definitive treatment by surgery. We selected this treatment period to provide sufficient time for aspirin to induce molecular changes in colorectal cancer tissues. The study was conducted following the Declaration of Helsinki, and the protocol was approved by the Clinical Investigation Ethics Committee of the Aragón Health Research Institute (Zaragoza, Spain) (EUDRACT number: 2018-002101-65; ClinicalTrials.gov Identifier: NCT03957902). All subjects provided written informed consent. The study was performed at Hospital Clínico Universitario Lozano Blesa (Zaragoza, Spain), and all biomarker assessments were carried out at CAST (“G. d’Annunzio” University, Chieti, Italy). The inclusion criteria were: age ≥18 and <80 years old; recent diagnosis (<48 h) of rectum or colon cancer, established by endoscopy and later confirmed by an anatomic-pathologic analysis and normal coagulation values. They were patients of the real-world setting. The exclusion criteria were allergy to aspirin or any other NSAID; CRC requiring neoadjuvant treatment within the 2 weeks following the beginning of aspirin treatment; previous use of aspirin, nonaspirin NSAIDs, other antiplatelet agents, corticosteroids or misoprostol within the 15 days before diagnosis and/or anticipation of a need for treatment with any of these drugs during the study period; history of peptic ulcer disease or active peptic ulcer or any other gastrointestinal disease that may be considered a contraindication to the use of aspirin, without the concomitant use of proton pump inhibitors; diagnosis of bleeding disorders; diagnosis of cancer (excluding non-melanoma skin cancer) within the previous 3 years; serious comorbidity, including respiratory, cardiac, hepatic and renal diseases; active smoking; pregnancy or breastfeeding; history of drug or alcohol abuse.

**FIGURE 2:**
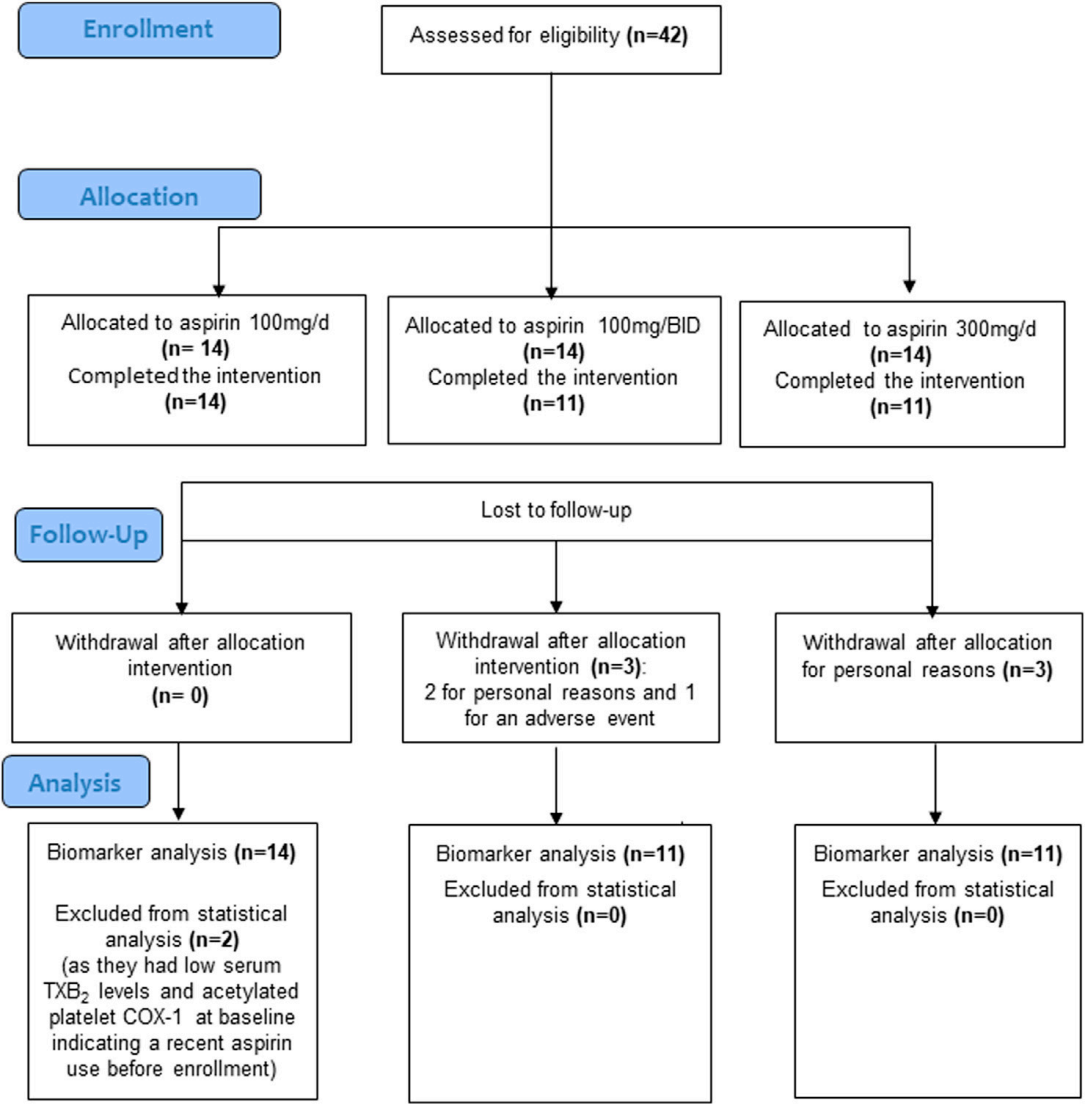
Flow diagram of the study.

The flow diagram of the study is reported in Figure 2. Two randomized patients to receive 100 mg/BID of aspirin withdrew for personal reasons and one for an adverse event (mild gastrointestinal bleeding). Three patients randomized to receive 300 mg/d of aspirin withdrew for personal reasons. However, two patients who completed the treatment with 100 mg/d were excluded from the statistical analyses because they had low serum TXB_2_ levels and acetylated platelet COX-1 at baseline, suggesting the use of aspirin before enrollment. The demographic and clinical characteristics of 34 CRC patients who completed the aspirin treatments and were included in the final biomarker analysis are reported in Table 1, and the tumor, node, metastasis (TNM) classification of malignant tumors in Table 2. The demographic, clinical, and TNM features of the three treatment groups are presented in Supplementary Tables S1, S2. Among the three groups, a significant difference was observed only for a lower percentage of hypertension in the 100 mg/d group as compared to that of the 100 mg BID group. The 300 mg/d group showed higher Alanine Aminotransferase (ALT) and gamma-glutamyltransferase values than the 100 mg/d group (Supplementary Table S1). The TNM classification and cancer localization did not differ significantly among the groups (Supplementary Table S2). As recommended by the national consensus of the Spanish Society of Pathology (SEAP) and the Spanish Society of Medical Oncology (SEOM) (García-Alfonso et al., 2012) we performed the analysis of retained or loss expression of the mismatch repair proteins (MMRs: MLH1, MSH2, MSH6, and PMS2) and the results are reported in Supplementary Table S3.

**TABLE 1:** The demographic and clinical characteristics of CRC patients completing Aspirin treatments and included in the biomarker analysis (values are reported as mean±SD).

CRC patients, *n* = 34	
Age, y	64.68 ± 9.47
Sex, Female, *n* (%)	8, (23.53)
BMI, kg/m^2^	27.93 ± 4.85
Systolic blood pressure (SBP), mmHg	129.1 ± 13.12
Diastolic blood pressure (DBP), mmHg	79.47 ± 10.73
Leukocytes (×10^3^/mm^3^)	7.39 ± 2.28
Platelets (×10^3^/mm^3^)	242.7 ± 70.74
Erythrocytes (×10^6^/mm^3^)	4.98 ± 0.39
Mean corpuscular hemoglobin concentration (MCHC), g/dL	33.39 ± 0.74
Hematocrit, %	43.45 ± 4.01
Haemoglobin, g/dL	14.51 ± 1.44
Aspartate transaminase (AST), IU/L	21.32 ± 6.98
Alanine aminotransferase(ALT), IU/L	17.76 ± 8.22
AST/ALT ratio	1.36 ± 0.59
Gamma-glutamyltransferase(GGT), IU/L	25.91 ± 16.76
Alkaline phosphatase(ALP), IU/L	78.41 ± 23.26
Total Bilirubin, mg/dL	0.737 ± 0.36
Glucose, mg/dL	105.5 ± 31.63
Creatinine, mg/dL	0.99 ± 0.24
Fibrinogen, mg/dL	516.6 ± 100.9
Urea, g/L	0.32 ± 0.11
Total cholesterol, mg/dL	188.9 ± 42.67
HDL cholesterol, mg/dL	53.15 ± 15.23
LDL cholesterol, mg/dL	117.1 ± 36.96
Triglycerides, mg/dL	93.82 ± 32.94
Comorbidity	
Diabetes, *n* (%)	9 (26.47)
Dyslipidemias, *n* (%)	14 (41.17)
Hypertension, *n* (%)	14 (41.17)
Concomitant drugs	
Statins, *n* (%)	13 (38.23)
ACE-inhibitors, *n* (%)	5 (14.70)
Sartans, *n* (%)	6 (17.64)
Hypoglycemics, *n* (%)	9 (26.47)
Diuretics, *n* (%)	7 (20.58)
Beta-adrenergic blocking agents, *n* (%)	1 (2.94)
Calcium channel blockers, *n* (%)	2 (5.88)

**TABLE 2:** TNM staging of colorectal tumors of the CRC patients completing Aspirin treatments and included in the biomarker analysis.

	CRC patients, *n* = 34
TNM classification	
T1, *n* (%)	0 (0)
T2, *n* (%)	5 (14.7)
T3, *n* (%)	26 (76.4)
T4, *n* (%)	3 (8.8)
N0, *n* (%)	13 (38.2)
N1, *n* (%)	8 (23.5)
N2, *n* (%)	13 (38.2)
N3, *n* (%)	0 (0)
M0, *n* (%)	28 (82.3)
M1, *n* (%)	5 (14.7)
Mx, *n* (%)	1 (2.9)
Location	
Cecum	2 (5.8)
Ascending colon	1 (2.9)
Transverse colon	3 (8.8)
Descending colon	1 (2.9)
Sigma	6 (17.6)
Rectum	16 (47)
Rectosigmoid junction	5 (14.7)

T = Tumor. T is based on the tumor size; generally, the number is between 1 and 4. A higher number means a larger tumor.

N = Lymph Nodes. A number after N indicates how many lymph nodes were found to have cancer; N0 (zero) means no cancer was found in the tested lymph nodes.

M = Metastasis. M0 (zero) means the cancer has not spread anywhere else in the body. M1 means that cancer has spread. Mx: Metastasis cannot be measured.

### 2.2 Study protocol

**FIGURE 3:**
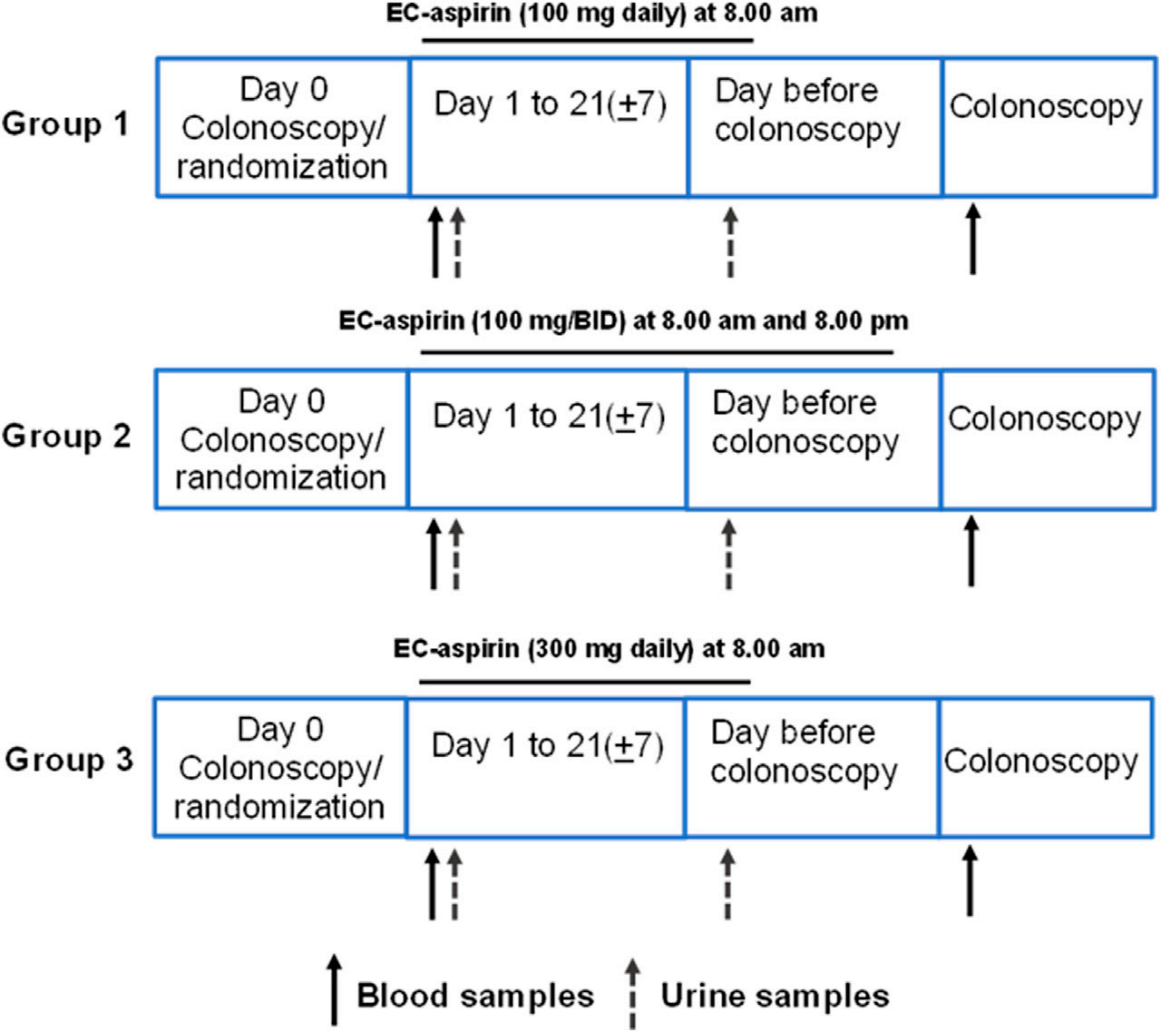
Study protocol.

The study protocol is outlined in Figure 3. On day 0 (baseline visit), patients attended the Clinical Research Unit and were diagnosed with CRC, and colorectal tissue biopsies were collected. After having signed the informed consent for the study, the following procedures were performed: medical history and physical examination, questions regarding alcohol and/or drugs of abuse, taking vital signs [blood pressure (BP), heart rate (HR)], laboratory assessment for tolerability and safety evaluation [urine pregnancy test (women of childbearing potential had to have a urine pregnancy test confirmed as negative within 24 h before the first dose of study and medication urine pregnancy test kits were provided), blood samples were collected to perform HIV, Hepatitis B and Hepatitis C tests, and hematological and biochemical tests]. Subjects were asked to return home and to come back to the Clinical Research Unit on Day 1 at 7 a.m., following an overnight fast of at least 10 h (only water was allowed to be taken). Patients collected overnight (at least 12 h) urine samples and brought them to the Clinical Research Unit on Day 1.

On day 1, at 7 a.m., fasted patients underwent a venous blood sample collection (baseline, pre-drug). Patients were randomized to receive enteric-coated (EC) aspirin 100 mg/d (at 8 a.m.) or, 100 mg BID (at 8 a.m. and 8 p.m.), or 300 mg/d (at 8 a.m.). They were administered the first dose of aspirin (100 mg or 300 mg) at 8 a.m., which was witnessed. The patients were instructed to take aspirin at home for up to 21 ± 7 days. Every five mornings, a researcher phoned to remind them of the drug administration.

On the day before the colonoscopy, at 7 a.m., patients returned to the Clinical Research Unit with a urine sample (24 h collection); then, they took witnessed aspirin administration (100 mg or 300 mg) at 8 a.m.; patients that were administered with aspirin 100 mg BID took the last dose at 8 p.m. The following day, at 7 a.m., patients returned to the Clinical Research Unit. Patients delivered the boxes for compliance assessment by aspirin pill counts. Pill count adherence was 100%. They underwent a venous blood sample collection at 8 a.m., and then all patients received a colonoscopy, and normal and neoplastic tissue samples were obtained (approximately 24 h after the last dose of aspirin 100 or 300 mg daily or 12 h after aspirin 100 mg BID).

### 2.3 Sample collections

Washed platelets were obtained from whole blood samples collected using sodium citrate 3.8% vacutainer tubes (Greiner Bio-One, Frickenhausen, Germany). Platelet-rich plasma (PRP) was obtained by centrifuging blood at 180 × g at 21°C for 16 min. Aliquots of PRP (500 mL) were centrifuged at 2000 × g for 15 min at room temperature, and platelets were washed and stored at −80°C until analysis, as previously described (Patrignani et al., 2014). For the assessment of plasma levels of ASA and its major metabolite salicylic acid (SA), peripheral blood samples were collected by using precooled vacutainer tubes containing sodium fluoride/potassium oxalate (Greiner Bio-One, Frickenhausen, Germany) to prevent the hydrolysis of ASA to SA. Additionally, sample handling was performed on ice to reduce the potential for hydrolysis of ASA (Patrignani et al., 2014).

Serum samples were obtained by collected whole blood by using tube vacutainers (Greiner Bio-One, Frickenhausen, Germany) and put them immediately into water bath at 37°C for 60 min. At the end of the incubation, serum was separated by centrifugation (10 min at 1700 × g, 4°C), collected and kept at −80°C (Patrono et al., 1980; Patrignani et al., 2014).

A colonoscopy was performed to collect colorectal biopsies of normal and cancerous tissue that were washed in PBS and immediately placed into a tube and frozen in liquid nitrogen. The specimens were stored at −80°C until the analysis (Patrignani et al., 2017). Some biopsy specimens used for prostanoid level evaluation were washed in PBS containing indomethacin (20 μM, Sigma-Aldrich, Milan) and immediately placed into a tube and frozen in liquid nitrogen, and stored at −80°C. Aliquots of overnight (at least 12 h) urine samples were immediately stored at −80°C.

### 2.4 Biomarker assessments

Figure 1 illustrates the biomarker strategy used in this study. The study’s primary endpoint was to evaluate the percentage of acetylation of COX-1 at Serine529 (AceCOX1) in cancerous recto-colonic tissues and platelets after the last dose of EC-aspirin. The aim was to test the hypothesis that higher doses of EC-aspirin lead to a higher percentage of AceCOX1 in cancerous recto-colonic tissue, while the percentage of AceCOX1 in washed platelets remains similar across different aspirin doses. The assessment of % acetylation of COX-2 (AceCOX-2) expressed in colorectal tissues by the different aspirin doses was a secondary endpoint. AceCOX-1 and AceCOX-2 were assessed by absolute quantification (AQUA) methods using LC-MS/MS (Li et al., 2014; Patrignani et al., 2014; Patrignani et al., 2017; Tacconelli et al., 2020). We evaluated the impact of EC-aspirin 100 mg/d, 100 mg/BID, and 300 mg/d on other secondary endpoints: 1) platelet COX-1 activity *ex vivo* by assessing serum TXB_2_ levels using a specific and validated immunoassay (Patrono et al., 1980; Patrignani et al., 2014); 2) COX-2 activity *in vivo* by assessing the urinary levels of primary enzymatic metabolites of PGE_2_ and PGI_2_ (prostacyclin), i.e., 11-α-hydroxy-9,15-dioxo-2,3,4,5-tetranor-prostane-1,20-dioic acid(PGE-M) and 2,3-dinor-6-keto-PGF_1α_(PGI-M), respectively (Dovizio et al., 2012), 3) COX-1 activity *in vivo* by assessing the urinary levels of major enzymatic metabolites of TXA_2_, i.e., 11-dehydro-TXB_2_ and 2,3-dinor-TXB_2_ (Catella and FitzGerald, 1987) and PGD_2_, i.e., 11,15-dioxo-9a-hydroxy-2,3,4,5-tetranorprostan-1,20-dioic acid(PGDM) (Song et al., 2008), using previously described LC-MS/MS techniques (Song et al., 2007; Patrignani et al., 2014; Lanas et al., 2023), 4) colorectal PGE_2_ and TXB_2_ levels by using validated immunoassays (Patrignani et al., 2014; Patrignani et al., 2017), 5) colorectal expression of genes encoding prostanoid biosynthesis enzymes and receptors, and EMT markers by qPCR as previously described (Sacco et al., 2019; Bruno et al., 2022; Lanas et al., 2023), 6) plasma levels of aspirin and salicylic acid(SA) by LC-MS/MS as previously reported (Patrignani et al., 2014).

### 2.5 Biochemical analysis

#### 2.5.1 Assessment of COX-isozyme acetylation in platelets and colorectal biopsies by AQUA (absolute quantification) methods

Unacetylated and acetylated forms of COX-1 were assessed in platelet pellets and colorectal biopsy specimens, while unacetylated and acetylated forms of COX-2 in colorectal biopsy specimens, as previously described. (Patrignani et al., 2014; Patrignani et al., 2017; Tacconelli et al., 2020) Briefly, platelet pellets and biopsy specimens were removed from the freezer, placed in 150 µL of lysis buffer prepared as previously described (Din et al., 2012; Patrignani et al., 2017) and completed with EDTA-free proteases inhibitors (Pierce, Waltham, MA, United States), 1 mM phenylmethylsulfonyl fluoride (PMSF; Sigma-Aldrich, Milan, Italy) and 62.5 U of benzonase nuclease (Calbiochem, San Diego, CA, United States). All samples (platelet and colorectal biopsies) were homogenized on ice for 30 s (2 cycles) by using TissueRuptor (Qiagen, Maryland, United States) and then sonicated on ice for 30 s (2 cycles), put on ice for 30 min, centrifuged at 1,690 g for 10 min at 4°C and stored at −80°C until the analysis. Bradford analysis was used for the quantitation of total proteins. Aliquots of sample homogenate were loaded onto a NuPAGE Tris-Acetate Gel (7%) (Life Technologies, Carlsbad, CA, United States). Regions of the gel corresponding to the molecular mass range from 60 to 85 kDa (which comprises COXs molecular mass) were excised and subjected to in-gel digestion with trypsin and GluC digestion for 2 h at 37°C (Tacconelli et al., 2020) to obtain a peptide of eight aminoacids containing the residue serine-529 (524-IGAPFSLK-531) for COX-1 and containing the residue serine-516 (511-VGAPFSLK-518) for COX-2 and their relative acetylated forms. (Patrignani et al., 2014; Tacconelli et al., 2020; Lanas et al., 2023) For absolute quantification AQUA peptides, i.e., the same endogenous peptides which were incorporated with stable isotopes ^13^C and ^15^N, IGAPFS [Leu(^13^C_6_;^15^N)]K and IG [Ala(^13^C_3_;^15^N)]PF [Ser(Ac)]LK for COX-1 and VGAPFS[Leu(^13^C_6_;^15^N)]K and VG[Ala(^13^C_3_;^15^N)]PF[Ser(Ace)]LK for COX-2, synthesized by Thermo Fisher Scientific (Waltham, MA, United States) (>97% purity), were added. The endogenous peptides (unacetylated and acetylated) and the corresponding AQUA peptides were assessed as previously described by using a platform ACQUITY UPLC I-Class/Xevo TQS micro-IVD System (Waters) (Tacconelli et al., 2020; Lanas et al., 2023). The absolute quantification is determined by comparing the abundance of the known AQUA internal standard peptides with the native peptides by LC-MS/MS.

#### 2.5.2 Assessment of urinary prostanoid metabolites

Overnight urine collections were obtained to assess urinary levels of 11-dehydro-TXB_2_ and 2,3-dinor-TXB_2_, PGE-M, PGI-M, and PGD-M by LC-MS/MS (Patrignani et al., 2017; Lanas et al., 2023). Urine samples (1 mL aliquot) were added with: tetranor PGEM-d_6_ (at final concentration of 25 ng/mL) (Cayman Chemical, Ann Arbor, Minn), tetranor PGDM-d_6_ (at final concentration of 25 ng/mL) (Cayman Chemical, Ann Arbor, Minn), 2,3dinor-6keto-PGF_1α_-d_9_ (sodium salt) (at final concentration of 10 ng/mL) (Cayman Chemical, Ann Arbor, Minn), 2,3dinor-TXB_2_-d_9_ (at final concentration of 10 ng/mL), 11-dehydro-TXB_2_-d_4_ (at final concentration of 10 ng/mL) (Cayman Chemical, Ann Arbor, Minn) as internal standards, and incubated at room temperature for 15 min; then formic acid (5 μL) is added. After 15 min of incubation, methoxyamine HCl (1 g/mL, 0.5 mL) (Sigma-Aldrich, St Louis, Mo) is added. Following 30 min of incubation at room temperature, urine samples were diluted to 10 mL with water adjusted to pH 3 with HCl and extracted with Number Strata x 33u polymeric reversed phase (30 mg/1 mL) (Phenomenex, Torrance, CA, United States) as previously described (Murphey et al., 2004). The eluate was evaporated, and the dried residue was resuspended in 100 μL mobile phase (10%acetonitrile in water), and 30 μL was injected into a ACQUITY UPLC I-Class/Xevo TQS micro-IVD System (Waters) equipped with an electrospray ionization source (ESI Z-Spray), under negative ionization conditions, as previously described (Lanas et al., 2023).

#### 2.5.3 Assessment of PGE_2_ and TXB_2_ levels in colorectal biopsies

Colorectal biopsies were washed in PBS containing indomethacin (20 μM, Sigma-Aldrich, Milan) to prevent prostanoid generation during tissue handling, and immediately placed in tubes and frozen in liquid nitrogen. For the homogenization, a buffer solution (400 μL, 0.05M Trizma base, pH = 7.4) containing indomethacin (20 μM) was added to each biopsy. Then, homogenization was performed on ice for 30 s, by using TissueRuptor (Qiagen, Germany). An aliquot of sample homogenate (50 μL) was used to quantify protein concentration by Bradford protein assay (Bio-Rad, Milan), while the residual aliquot of homogenate sample (350 μL), after extraction, was analyzed for PGE_2_ and TXB_2_ levels. For the extraction of the prostanoids, we added ^3^H-6-ketoPGF_1α _(specific activity, 140Ci/mmol, Perkin Elmer Inc., Milan, Italy) to the homogenate sample (for recovery evaluation) and then 1 mL of methanol (Carlo Erba Reagents, Milan, Italy) was added and samples were vortexed for 1 min, put on ice for 5 min and then ice-cold deionized water was added to a final methanol concentration of 10%; after vortexing for 1min, samples were centrifuged at 3,000 rpm for 10 min at 4°C and supernatants were collected. After adjusting the pH to 4 with formic acid, samples were loaded on Sep-Pack^®^ Plus C18 cartridges (Waters Associates, Milford, MA), conditioned with methanol and deionized water, after washing with deionized water and hexane (Carlo Erba Reagents), and finally eluted with 10 mL of ethyl acetate (Carlo Erba Reagents). After evaporation of ethyl acetate to dryness, the extracts were reconstituted with 0.250 mL of Tris-phosphate buffer (0.02M, pH 7.4) and the levels of TXB_2_ and PGE2 (2 in pedix) were assessed by specific and previously validated immunoassays (Patrignani et al., 2014; Patrignani et al., 2017) while recovered [^3^H]-1a are as pedix was measured by a β-counter (Tri-Carb 2100 TR Perkin Elmer).

#### 2.5.4 Assessment of gene expression by qPCR in colorectal biopsies

Colorectal biopsies were homogenized with TissueRuptor (Qiagen, Maryland, United States); total RNA was extracted using Pure link RNA Mini kit (Life Technologies, Carlsbad, CA, United States) according to the manufacturer’s protocols. Total RNA (2 μg) was treated with DNAse kit (Fermentas, St. Leon-Rot, Germany) and reverse-transcribed into cDNA using Iscript cDNA Synthesis Kit (Bio-Rad, Milan, Italy) according to the manufacturer’s protocols. One hundred nanograms of cDNA were used for the reaction mixture (Lanas et al., 2023). For the assessment of gene expression in colorectal samples of CRC patients, the amplification of *PTGS1* (protein name: COX-1), *PTGS2* (protein name COX-2), *HPGD* (protein name: 15-PGDH), *PTGES* (protein name: prostaglandin E synthase, also called mPGES-1), *PTGES3* (protein name: Prostaglandin E synthase 3, also called cPGES), *TBXAS1* (protein name: TXA_2_ synthase), *PTGER4* (protein name: EP4), *TBXA2R* (protein name: TP), *PTGDR* (protein name: DP), *PTGDR2* (protein name: CRTH2), *FN1* (protein name: fibronectin), *CDH1* (protein name: E-cadherin), *VIM* (protein name: vimentin), *TWIST1* (protein name: Twist-related protein 1), *RAC1* (protein name: ras-related C3 botulinum toxin substrate 1) and *GAPDH* was performed using TaqMan gene expression assays [Hs00377726, Hs00153133, Hs00960586, Hs01115610, Hs00832847, Hs01022706, Hs00168761, Hs00169054, HS00235003, Hs01867513, Hs00365052, Hs01023894, Hs00185584, Hs01675818, Hs01902432 and Hs99999905, respectively (Applied Biosystems, Foster City, CA)] according to the manufacturer’s instructions using a 7900HT Real-Time PCR system (Applied Biosystems, Foster City, CA).

#### 2.5.5 Assessment of serum TXB_2_

Serum TXB_2_ levels were assessed using a validated enzyme immunoassay (EIA) kit (Cayman Chemical, Ann Arbor, United States) (Patrignani et al., 2014).

#### 2.5.6 Assessment of plasma levels of acetylsalicylic acid and salicylic acid

Plasma samples, combined with d_4_-acetylsalicylic acid and d_4_-salicylic acid (Santa Cruz Biotechnology, Dallas, TX, United States), were extracted and then analyzed by using ACQUITY UPLC I-Class/Xevo TQS micro-IVD System (Waters) equipped with a Z-Spray ESI source, operating in negative ion mode, as previously described (Patrignani et al., 2014).

#### 2.5.7 Immunohistochemical analysis of the mismatch repair proteins (MMRs)

Immunohistochemistry staining for the MMRs was performed by using the DAKO OMNIS automated staining system (Agilent, CA, United States) on representative paraffin-fixed colorectal cancer tissue blocks. All the antibodies were ready-to-use monoclonal mouse antibodies provided in liquid form in a buffer containing stabilizing protein and 0.015 mol/L sodium azide, including MLH1 (clone ES05), MSH2 (clone FE11), MSH6 (clone EP49), and PMS2 (clone EP51); monoclonal antibodies were from Dako (Glostrup, Denmark). All staining procedures were performed according to the manufacturer’s recommendations (Dako). The expression of MMR proteins in the nuclei of tumor cells was evaluated by pathologists, after confirming appropriate staining of the nuclei in internal positive controls within the tissue sections. The assessment included evaluating retained or lost expression for each MLH1, MSH2, MSH6, and PMS2 protein (Denčić et al., 2023).

### 2.6 Statistical analysis

Data were assessed for the normality test by D’Agostino-Pearson. Normal or lognormal (transformed to logarithms) distributed data were reported as mean ± SD and parametric tests made statistical comparisons. Simple and multiple linear regression analyses of the results were performed. A probability value of *p* < 0.05 was considered statistically significant. Statistical analysis was performed using GraphPad Prism Software(version 10.00 for Mac, RRID:SCR_002798, San Diego, CA, United States). Student’s t-test was used to compare the means of two independent groups; ANOVA followed by Tukey’s or Šídák’s multiple comparisons test was used to compare the means of more than two independent groups. The differences in the distribution of categorical variables were assessed by the chi-square test. The specific test used was reported in the legends of each figure. The investigator responsible for data analysis was blinded to experimental group results. The study’s primary endpoint was the assessment of COX-1 acetylation in platelets *vs.* nucleated cells of the colorectal cancer tissue after dosing with EC-aspirin to address the hypothesis that the administration of EC-aspirin would cause maximal acetylation of platelet COX-1 (approximately 70%) (comparable at each dose) while causing lower acetylation (30% and 50%) of COX-1 in colorectal tissue after dosing with EC-aspirin 100 mg/d and 300 mg/d, respectively. Based on a previous study performed on healthy subjects, we calculated an intersubject coefficient of variation (CV) of platelet AceCOX-1 of 10% at 24 h after EC-aspirin 100 mg/d administration for 7 days (Patrignani et al., 2014). Assuming this intersubject CV, a sample size of 10 patients has a 95% power to detect a difference between means of 17.10 or higher with a significance level (alpha) of 0.05 (two-tailed). Considering a possible patient dropout rate of 0.3, we selected a sample size of 14 individuals.

## 3 Results

### 3.1 Baseline features of CRC patients enrolled in the clinical study

#### 3.1.1 CRC patients’ background, TNM staging, retained MMR expression, and KRAS, NRAS, and BRAF mutations

A total of 34 CRC patients completed the treatment with aspirin and were included in the biomarker assessment study (Figure 2). As shown in Table 1, the CRC patients were mostly male (76%) and had comorbidities such as diabetes, dyslipidemia, and hypertension (26%, 41%, and 41%, respectively). They had not yet received the first therapy for their newly diagnosed CRC, but appropriate comorbidity treatments were given, and the drugs are listed in Table 1. According to the TNM staging system, most of the tumors were non-metastatic (M0 in 82% of patients), and the size was classified as T3 (76% of patients) (Table 2). The number of lymph nodes with tumors (N) ranged from 0 to 2 within the patient group (Table 2). In the majority of patients (79%), cancer was found in the rectum, sigma, and rectosigmoid junction. In 29 CRC patients, we evaluated the loss or retained expression of MMR proteins in CRC tissue assessed through immunohistochemistry, and we found that only one patient had a loss of protein expression of PMS2 and MLH1 (Supplementary Table S3).

#### 3.1.2 Receptor and enzyme expression for prostanoid pathways in CRC patients related to the systemic, platelet, and tumor tissue prostanoid biosynthesis

**FIGURE 4:**
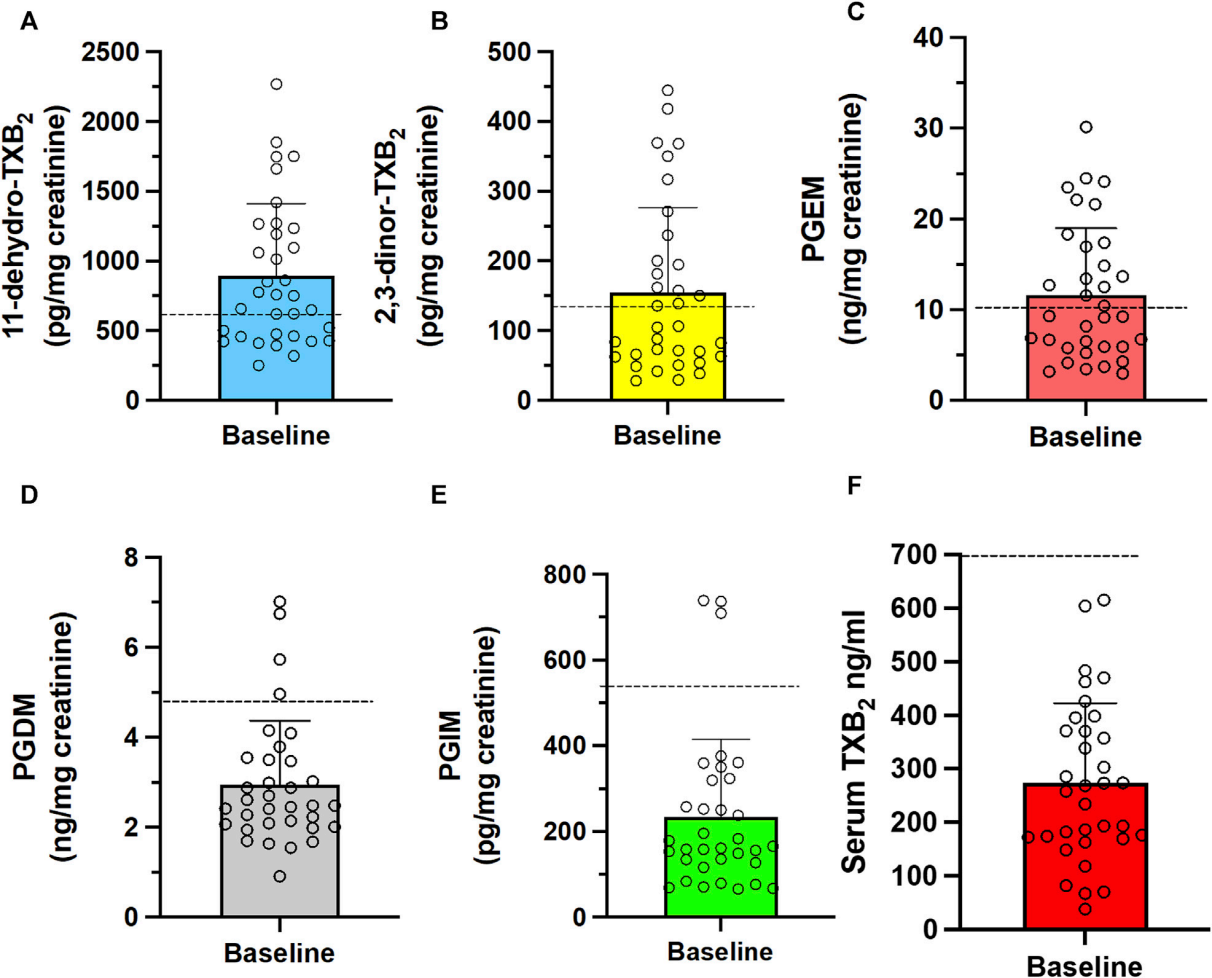
Baseline systemic biosynthesis of prostanoids and baseline values of platelet TXB_2_ biosynthesis in CRC patients. Urinary levels of the primary enzymatic metabolites of **(A,B)** TXA_2_, i.e., 11-dehydro-TXB_2_ and 2,3-dinor-TXB_2_; **(C)** PGI_2_, i.e., 2,3-dinor-6-keto-PGF_1α_ (PGIM); **(D)** PGE_2_, i.e., PGEM; **(E)** PGD_2_, i.e., PGDM and **(F)** serum TXB_2_, a capacity marker of platelet COX-1 activity. Individual data are reported as scatter dot plots and mean + SD, *n* = 34. The horizontal dashed lines show the cutoff values previously detected in healthy subjects (mean + 2SD) (Patrignani et al., 2014; Patrignani et al., 2017; Lanas et al., 2023).

**FIGURE 5:**
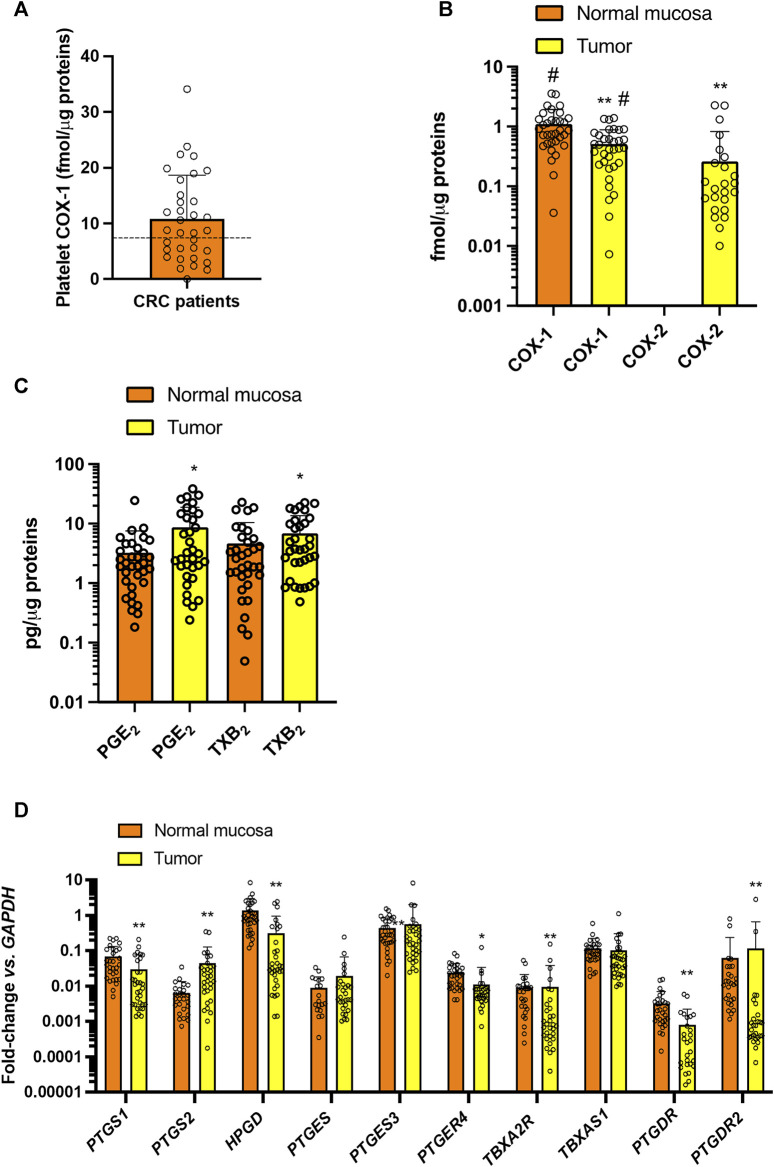
Baseline values of COX-1 expression in platelets and prostanoid biosynthesis and expression of enzymes and receptors of prostanoid pathways in colorectal biopsies. **(A)** Platelet COX-1 levels quantified by LC-MS/MS in washed platelets. Individual data are reported as scatter dot plots and mean + SD (*n* = 34). The horizontal dashed lines show the cutoff values previously detected in healthy subjects (mean + 2SD) (Patrignani et al., 2014; Patrignani et al., 2017). **(B)** COX-1 and COX-2 levels were quantified by LC-MS/MS in normal and colorectal cancer biopsy; individual data are reported as scatter dot plots and mean + SD (*n* = 34) and analyzed by paired t-test, normal mucosa *vs.* tumor for each COX-isozyme, and one-way ANOVA and Tukey’s *post hoc* tests, **p < 0.01 *vs.* normal mucosa, ^#^ *p* < 0.01 *vs.* all other conditions. COX-2 was undetectable in all normal colorectal tissues and in 10/34 patients’ CRC biopsies. **(C)** PGE_2_ and TXB_2_ levels were assessed in normal and CRC biopsy extracts by immunoassays; data are reported as scatter dot plots with mean + SD, *n* = 34; data did not pass the normality test and were log-transformed and analyzed by paired t-test normal mucosa *vs.* tumor for each prostanoid; *p < 0.05 *vs.* normal mucosa. **(D)** *PTGS1* (COX-1), *PTGS2* (COX-2), *HPGD* (15PGDH), *PTGES* (mPGES-1), *PTGES3* (cPGES), *PTGER4* (EP4), *TBXA2R* (TP), *TBXAS1* (TXS), *PTGDR* (DP1), *PTGDR2* (CRTH2), *PTGER4* (EP4) and *GAPDH* were assessed by qPCR in normal and colorectal cancer tissue samples; gene expression is reported as fold change *vs. GAPDH*; all data are reported as scatter dot plots with mean + SD, *n* = 26–32 (in some samples the genes were undetectable). Since data did not pass the normality test were transformed into logarithms. All data were analyzed by two-way ANOVA followed by Šídák’s multiple comparisons test; *p < 0.05, **p < 0.01 *vs.* normal mucosa.

In the 34 CRC patients who completed the study, the baseline systemic biosynthesis of TXA_2_ was assessed by measuring the urinary levels of the two major enzymatic metabolites, 11-dehydro-TXB_2_ and 2,3-dinor-TXB_2_ (Figures 4A, B) (Catella and FitzGerald, 1987). Additionally, the systemic biosynthesis of PGE_2_, PGD_2_, and PGI_2_ was assessed by evaluating the levels of their urinary enzymatic metabolites PGEM, PGDM, and PGIM, respectively (Figures 4C–E) (Catella and FitzGerald, 1987; Song et al., 2007; Song et al., 2008; Dovizio et al., 2012; Lanas et al., 2023). The levels of TXB_2_ produced during whole blood clotting at 37°C for 1 h (serum TXB_2_) were also measured (Figure 4F) as a marker of the maximal capacity of platelet COX-1 to generate TXA_2_ *ex vivo* (Patrono et al., 1980; Patrignani et al., 2014). We evaluated the levels of COX-1 in circulating platelets (Figure 5A).

In colorectal cancer biopsies of CRC patients, the protein levels of COX-1 were slightly lower than in normal mucosa of the same individuals (Figure 5B). COX-2 protein was undetectable in normal colorectal tissue but was found in 24/34 (70%) of the cancerous tissues analyzed (Figure 5B). Enhanced levels of PGE_2_ and TXB_2_ were found in CRC tissue *vs.* normal mucosa (Figure 5C).

The gene expression of COX-1 (gene name *PTGS1*) and 15-PGDH (gene name *HPGD*), involved in the metabolism of PGE_2_ to the less active 15-keto-PGE_2_ (Tai et al., 2002) was significantly downregulated in CRC tissues *vs.* normal mucosa (Figure 5D). A low gene expression of COX-2 (gene name *PTGS2*) was detected in 80% of normal mucosa samples analyzed. *PTGS2* was detected in 94% of colorectal cancers, and the expression was significantly enhanced *vs.* normal mucosa (Figure 5D). The downstream synthases involved in PGE_2_ and TXA_2_ biosynthesis were not significantly different in cancer tissues *vs.* normal mucosa. In contrast, the gene expression of the prostanoid receptors *PTGER4* (EP4), *TBXA2R* (TP), *PTGDR* (DP1), and *PTGDR2* (CRTH2) were significantly downregulated in CRC biopsies *vs.* normal mucosa (Figure 5D).

We performed simple linear regression of the biomarkers analyzed (Supplementary Figures S1A, B). Urinary 11-dehydro-TXB_2_ (TXM) was significantly correlated with 2,3-dinor-TXB_2_, PGDM, serum TXB_2_, platelet COX-1, and colorectal tumor COX-2 expression. Urinary PGEM was significantly correlated with platelet COX-1, tumor COX-2 (protein and mRNA), normal mucosa gene expression of cPGES, 15-PGDH, and EP4. Urinary PGIM was correlated with PGEM, serum TXB_2,_ and platelet COX-1. The gene expression of TXA_2_ receptors (TP) in tumors showed a significant correlation with the gene expression of COX-1, COX-2, 15-PGDH, mPGES-1, TXAS, EP4, and DP1 in tumors (Supplementary Figures S1A, B). These findings suggest that the TP receptors may have a part in controlling the gene expression of other prostanoid receptors and enzymes for prostanoid metabolism in colorectal tumors.

By carrying out multiple linear regression analysis of biomarkers and clinical laboratory data, we found that urinary 11-dehydro-TXB_2_ was predicted by urinary PGDM, platelet COX-1, tumor mRNA COX-2, and MCHC (mean corpuscular hemoglobin concentration) (Supplementary Table S4A). Urinary PGEM was predicted by PGIM, platelet COX-1, tumor mRNA COX-2 and 15-PGDH, and normal tissue PGE_2_ levels (Supplementary Table S4B). Overall, these results suggest the contribution of platelet and tumor COX-isozymes in the systemic biosynthesis of TXA_2_ and PGE_2_ in CRC patients.

Urinary PGDM was significantly associated with 11-dehydro-TXB_2_, blood pressure (BP), glucose, and LDL (Supplementary Table S5A). Urinary PGDM is a marker of the systemic biosynthesis of PGD_2_, which modulates vascular, platelet, and leukocyte function *in vitro* (Song et al., 2008). Our data may suggest that PGDM measurement can predict the CV risk of CRC patients.

### 3.2 Effects of aspirin doses (100 mg/d, 100 mg/BID, and 300 mg/d) on platelets and systemic biomarkers of prostanoid biosynthesis

**FIGURE 6:**
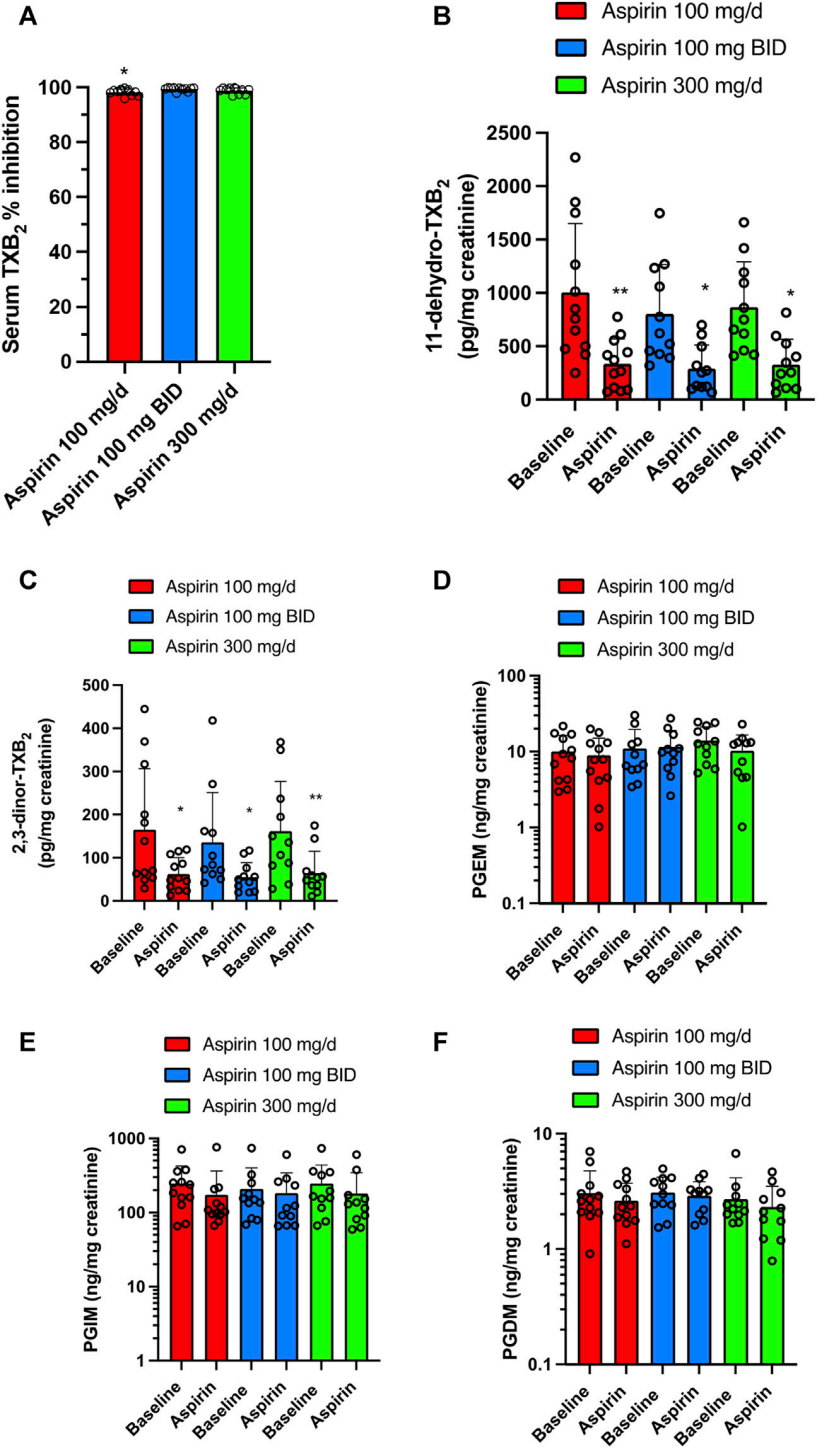
Effects of different doses of Aspirin (100 mg/d, 100 mg BID, and 300 mg/d) on platelet COX-1 activity and systemic biosynthesis of prostanoids in CRC patients. **(A)** Effect of Aspirin doses on platelet COX-1 activity of CRC patients; serum was obtained by allowing whole blood samples to clot for 1 h at 37°C, and a validated immunoassay assessed TXB_2_. All values are shown as scatter dot plots with mean + SD (*n* = 11, 12). *p < 0.05 *vs*. Aspirin 100 mg BID using one-way ANOVA and Tukey’s *post hoc* test. **(B–F)** Effects of different doses of Aspirin on the systemic biosynthesis of TXA_2,_ PGE_2_, PGI_2_, and PGD_2_ by assessing their primary urinary enzymatic metabolites [11-dehydro-TXB_2_ **(B)** and 2,3-dinor-TXB_2_ **(C)**, PGEM **(D)**, PGIM **(E)** and PGDM **(F)**] by LC-MS/MS. All values are shown as scatter dot plots with mean + SD (*n* = 11, 12). **p < 0.01, *p < 0.05 *vs.* baseline values using one-way ANOVA and Tukey’s *post hoc* test. For PGEM, PGIM, and PGDM, since data did not pass the normality test were transformed into logarithms and were analyzed by one-way ANOVA and Tukey’s *post hoc* test.

Daily aspirin at 100 mg/d caused virtually complete inhibition of serum TXB_2_, i.e., 98.26% ± 1.11%, which is appropriate to cause inhibition of platelet function *in vivo* (Reilly and FitzGerald, 1987; Patrono, 1994; Patrignani et al., 2014), and was not significantly different from that found with aspirin 300 mg/d (98.83% ± 1.05%) (Figure 6A). The administration of aspirin, 100 mg/BID, was associated with 99.41% ± 0.63% inhibition of serum TXB_2_ that showed a statistically significant difference (*p* < 0.05) only when compared to aspirin 100 mg/d (Figure 6A).

Aspirin 100 mg/d, 100 mg/BID, and 300 mg/d significantly and comparably reduced the urinary levels of 11-dehydro-TXB_2_ (66.46, 63.72, and 62.2%, respectively) (Figure 6B; Supplementary Figure S2A). Also, the other urinary enzymatic metabolite of TXA_2_, 2,3-dinor-TXB_2_, was significantly reduced by the 3 aspirin doses (62.17, 59.49, and 60.07%, respectively) (Figure 6C; Supplementary Figure S2B). The three doses of aspirin did not significantly affect the urinary levels of PGEM, PGIM, and PGDM (Figures 6D–F; Supplementary Figures S2C–E).

### 3.3 Effects of varying doses of aspirin on the acetylation of COX-isozymes

**FIGURE 7:**
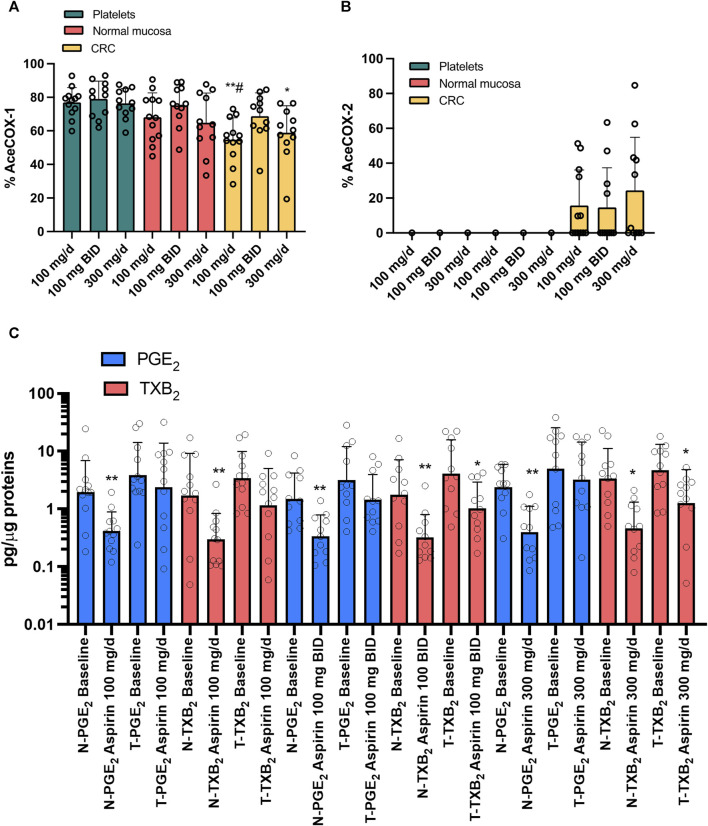
Effects of treatment with different doses of Aspirin (100 mg/d, 100 mg BID, and 300 mg/d) in CRC patients on the % acetylation of COX-1 **(A)** and COX-2 **(B)** in isolated platelets, normal and cancerous mucosa and on prostanoids in both normal and cancerous colorectal tissue. The extent of COX-1 (%AceCOX-1) **(A)** and COX-2 (%AceCOX-2) **(B)** acetylation was assessed in isolated platelets and both normal and cancerous colorectal tissue by LC-MS/MS. All values are shown as scatter dot plots with mean + SD (*n* = 11, 12) and analyzed by one-way ANOVA and Tukey’s *post hoc* test. Aspirin 100 mg/d: **p < 0.01 *vs.* platelets, ^#^ *p* < 0.05 vs. normal mucosa; Aspirin 300 mg/d: *p < 0.05 *vs.* platelets. **(C)** PGE_2_ and TXB_2_ levels were assessed by immunoassays in extracts of colorectal biopsies collected from normal (N) and tumor (T) tissue of patients with CRC at baseline and after treatment with Aspirin 100 mg/d (*n* = 12), 100 mg BID (*n* = 11) and 300 mg/d (*n* = 11). The values are pg/μg of proteins. All values are shown as scatter dot plots with mean + SD (*n* = 11, 12). Since data did not pass the normality test, they were transformed into Log_10_ and analyzed by one-way ANOVA followed by Tukey’s *post hoc* test; *p < 0.05, **p < 0.01 *vs.* normal mucosa at each experimental condition.

In platelets, colorectal normal, and tumor tissue, there were no significant changes in the extent of COX-1 acetylation among the varying doses of aspirin (Figure 7A). At aspirin 100 mg/d, the % AceCOX-1 of cancer tissue was significantly lower than that found in platelets and normal mucosa. At aspirin 100 mg/BID, % AceCOX-1 of cancer tissue was not significantly different from the values detected in platelets and normal mucosa. When taking 300 mg/d of aspirin, cancer tissue % AceCOX-1 was significantly lower than in platelets but not in normal mucosa (Figure 7A). However, 300 mg/d and 100 mg/BID of aspirin caused comparable acetylation of COX-1 in the CRC tissue.

We found that only 33, 36% and 45% of patients had acetylated COX-2 (% AceCOX-2 >10) in their tumor tissues when taking aspirin at 100 mg/d, 100 mg/BID, and 300 mg/d doses, respectively (i.e., 4 out of 12, 4 out of 11, and 5 out of 11 CRC patients, respectively) (Figure 7B). The average % AceCOX-2 in these patients was 42.14, 40.31, and 53.14%, respectively. These values were not statistically different.

### 3.4 Impact of varying doses of aspirin on intestinal prostanoid levels

We measured the levels of PGE_2_ and TXB_2_ in both normal colorectal mucosa and tumor tissues of CRC patients before and after administering the three doses of aspirin (Figure 7C). In normal colorectal mucosa, aspirin significantly reduced PGE_2_ and TXB_2_, even if partially, at all doses. In cancer tissue, aspirin 100 mg/d did not significantly affect the levels of PGE_2_ and TXB_2_. However, when taken at 100 mg/BID or 300 mg/d, PGE_2_ levels remained unaffected, while TXB_2_ levels were significantly reduced, although incompletely (Figure 7C). The two doses of aspirin caused a comparable reduction of TXB_2_ levels in CRC tissue.

### 3.5 Effects of varying doses of aspirin on colorectal cancer gene expression

**FIGURE 8:**
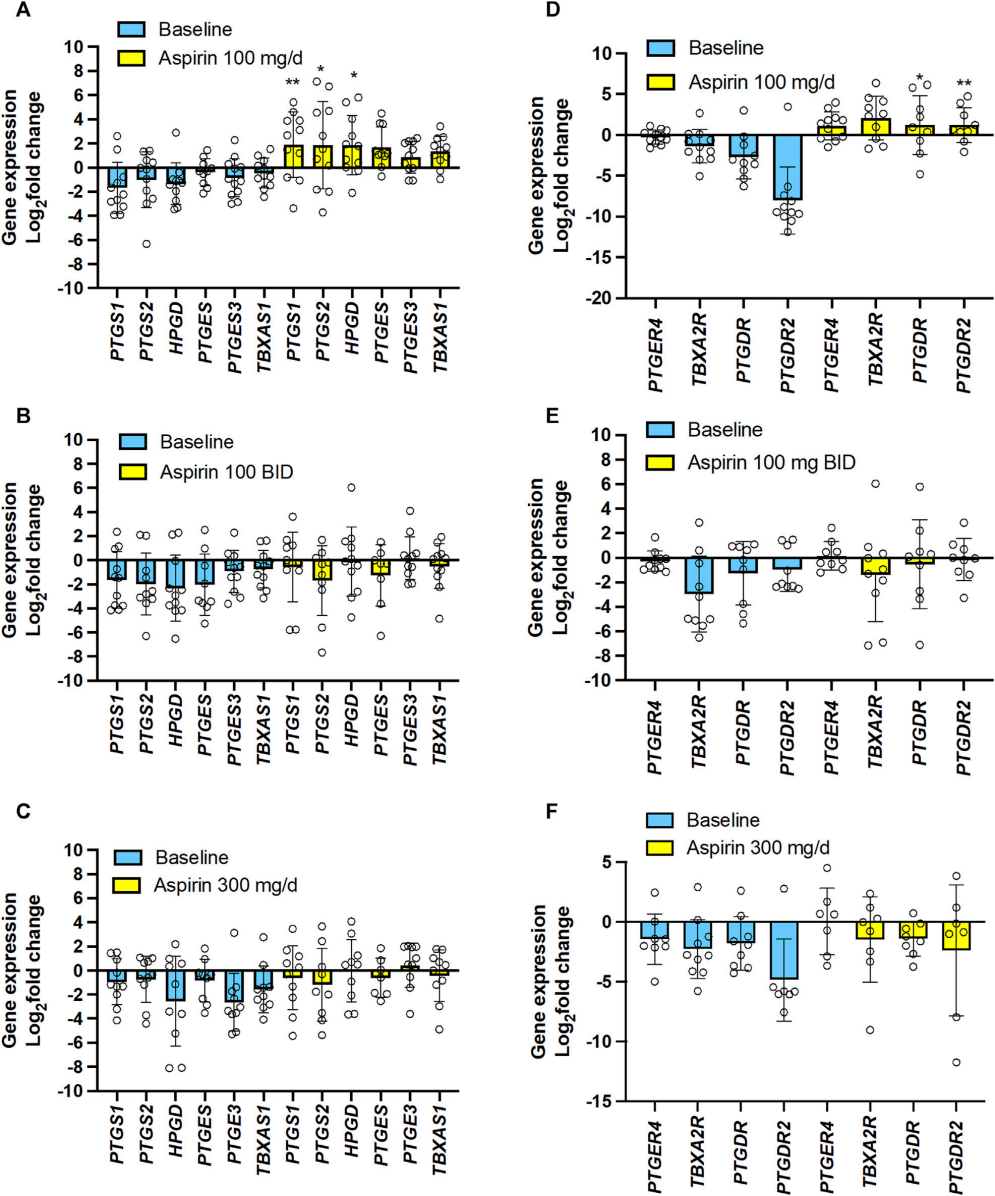
Effects of varying doses of Aspirin on colorectal cancer mucosa expression of genes encoding for COX-isozymes, downstream synthases, 15-PGDH, and prostanoid receptors. The mRNA levels of *PTGS1* (protein name: COX-1), *PTGS2* (protein name COX-2), *HPGD* (protein name: 15-PGDH), *PTGES* (protein name: prostaglandin E synthase, also called mPGES-1), *PTGES3* (protein name: Prostaglandin E synthase 3, also called cPGES), *TBXAS1* (protein name: TXA2 synthase), *PTGER4* (protein name: EP4), *TBXA2R* (protein name: TP), *PTGDR* (protein name: DP), *PTGDR2* (protein name: CRTH2) and *GAPDH* were assessed by qPCR in colorectal biopsies before and after Aspirin treatment. **(A,D)** Aspirin 100 mg/d, **(B,E)** Aspirin 100 mg BID, **(C,F)** Aspirin 300 mg/d. Gene expression in colorectal cancer biopsies was evaluated by qPCR and normalized to GAPDH mRNA levels. The yellow bars indicate the baseline (predrug) log_2_ fold change of gene expression compared to the average values found in all patients. Meanwhile, the pale blue bars display the log_2_ fold change of gene expression caused by Aspirin *vs.* the baseline values. All values are shown as scatter dot plots with mean ± SD (*n* = 7–12) and analyzed by one-way ANOVA followed by Tukey’s *post hocpost hoc* test. **(A,D)** *p < 0.05, **p < 0.01 *vs*. the same gene at baseline.

We studied the effect of the 3 doses of aspirin (100 mg/d, 100 mg/BID, and 300 mg/d) on CRC tissue expression of genes encoding COX-1 and COX-2 and downstream prostanoid synthases (Figures 8A–C) and prostanoid receptors *vs*. baseline (Figures 8D–F). Aspirin 100 mg/d was associated with significantly enhanced gene expression of COX-1 and COX-2, and 15-PGDH (Figure 8A), and the PGD_2_ receptors DP1 and CRTH2 (Figure 8D); TP receptor expression was also enhanced in most individuals (Figure 8D). The expression of these genes was not changed *vs.* baseline after taking 100 mg/BID or 300 mg/d of aspirin (Figures 8B, C, E, F).

**FIGURE 9:**
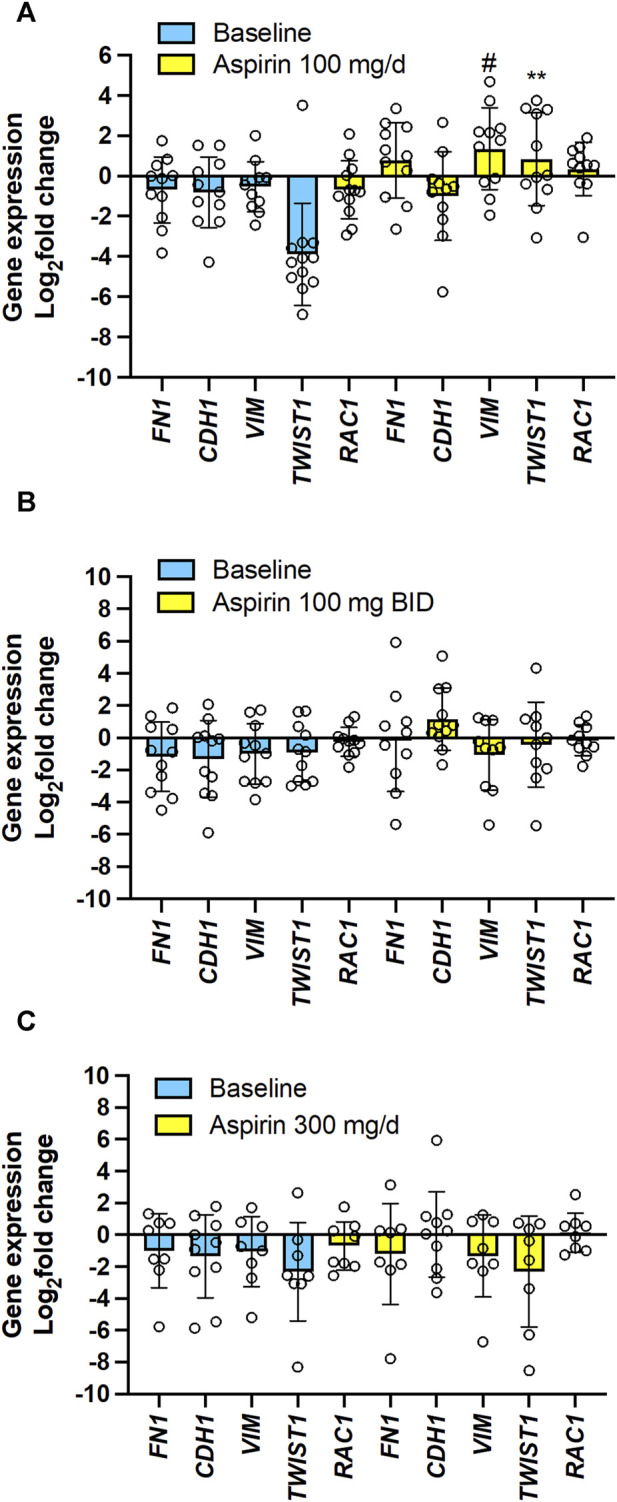
Effects of varying doses of Aspirin on colorectal cancer mucosa expression of genes encoding for EMT marker genes. The mRNA levels of *FN1* (protein name: fibronectin), *CDH1* (protein name: E-cadherin), *VIM* (protein name: vimentin), *TWIST1* (protein name: Twist-related protein 1), *RAC1* (protein name: ras-related C3 botulinum toxin substrate 1) and *GAPDH* were assessed by qPCR in colorectal biopsies before and after Aspirin treatment. **(A)** Aspirin 100 mg/d, **(B)** Aspirin 100 mg BID, **(C)** Aspirin 300 mg/d. Gene expression in colorectal cancer biopsies was evaluated by qPCR and normalized to GAPDH mRNA levels. The yellow bars indicate the baseline (predrug) log_2_ fold change of gene expression compared to the average values found in all patients. Meanwhile, the pale blue bars display the log_2_ fold change of gene expression caused by Aspirin *vs.* the baseline values. All values are shown as scatter dot plots with mean ± SD (*n* = 7–11) and analyzed by one-way ANOVA followed by Tukey’s *post hoc* test. **(A)** ^#^ *p* = 0.059, **p < 0.01 *vs*. the same gene at baseline.

The EMT gene expression markers analysis showed that 100 mg/d of aspirin was associated with significantly increased *Vim* and *TWIST1* (Figure 9A). However, this was not observed with the other two aspirin doses (Figures 9B, C).

The simple linear regression analysis of the % reduction of TXB_2_ levels and log_2_(fold change) of the expression of the 15 genes in colorectal tumor tissues by aspirin (all doses’ data were examined together) *vs.* the values assessed at predrug (baseline) showed that the % reduction of tumor TXB_2_ by aspirin inversely correlated with log_2_(fold change) of *PTGS2* (COX-2), *TWIST1* (twist-related protein 1) and *VIM* (vimentin) expression (Supplementary Figures S3A, B). Log_2_(fold change) of TP receptor expression by aspirin was inversely correlated with *CDH1* (E-cadherin) and positively correlated with *FN1* (fibronectin) and *VIM* (Supplementary Figures S3A, B).

## 4 Discussion

Evidence from clinical and experimental findings suggests that aspirin can act as an anticancer agent (Patrignani and Patrono, 2016). However, the precise mechanism of action is yet to be fully understood, and there is an ongoing debate regarding the appropriate dose. We assessed direct biomarkers of aspirin action consisting of the analysis of the extent of acetylation at Serine 529 and Serine 516 of COX-1 and COX-2 (Patrignani et al., 2014; Patrignani et al., 2017; Tacconelli et al., 2020), respectively, in platelets and colorectal tissues of CRC patients randomized to receive aspirin 100, 300 mg/d, or 100 mg/BID for ∼3 weeks. These effects were related to prostanoid biosynthesis in different body compartments by assessing biomarkers of their systemic biosynthesis (Song et al., 2007). In the CRC tissues, we compared the dose-response of aspirin inhibitory effects on TXA_2_ and PGE_2_ biosynthesis with the changes in expression profiles of COX-2 and EMT marker genes involved in cancer cell initiation, growth, and metastasis (Wang and Dubois, 2010; Brabletz et al., 2018). Our findings indicate that all three aspirin doses have comparable effects in inhibiting platelets from contributing to the systemic biosynthesis of TXA_2_ in CRC. However, taking aspirin 300 mg/d or 100 mg/BID proved to be more effective than 100 mg/d in reducing TXB_2_ levels and restraining the expression of tumor-promoting genes in CRC tissue. These findings indicate that a higher dose of aspirin or more frequent daily dosing can improve anticancer efficacy over 100 mg/d because of the concurrent inhibition of the generation of TXA_2_ in the colorectal cancer tissue and the platelets. The three aspirin doses did not significantly impact the biosynthesis of PGE_2_ *in vivo* evaluated by assessing urinary PGEM and PGE_2_ in colorectal tissue, presumably explained by the variable and incomplete impact of aspirin, at any dose, to acetylate COX-2 at Serine 516 in CRC tissue. COX-2 pathway is the major source of urinary PGEM (Dovizio et al., 2012).

Notably, in normal mucosa, all aspirin doses caused a profound extent of COX-1 acetylation at Serine 529 associated with reduced PGE_2_ biosynthesis, considered a cytoprotective pathway for the gastrointestinal system (Patrignani and Patrono, 2015). These effects and the comparable antiplatelet action predict a similar risk of bleeding associated with 100 mg/d and 300 mg/d of aspirin; this is confirmed by the results of the ADAPTABLE trial (Jones et al., 2021).

In patients with CRC, the baseline urinary levels of the primary TXA_2_ and PGE_2_ metabolites, 11-dehydro-TXB_2_ and 2,3-dinor-TXB_2_ and PGEM, were higher than the upper cutoff values of these metabolites previously found in healthy individuals (Patrignani et al., 2014; Patrignani et al., 2017; Lanas et al., 2023) (Figures 4A–C). Enhanced urinary 11-dehydro-TXB_2_ was previously reported in CRC patients (Sciulli et al., 2005; Joharatnam-Hogan et al., 2023) and has been interpreted as an index of platelet activation. We found that CRC patients also have elevated levels of COX-1 protein in their platelets *vs.* those detected in previous studies in healthy subjects (Patrignani et al., 2014; Patrignani et al., 2017) (Figure 5A). The significant correlation of urinary 11-dehydro-TXB_2_ with serum TXB_2_ and platelet COX-1 levels confirms the platelet contribution to 11-dehydro-TXB_2_ formation *in vivo* in CRC patients. However, there was a significant association between the levels of urinary 11-dehydro-TXB_2_ and the expression of tumor COX-2. Thus, in addition to the platelet, colorectal tumor TXB_2_ contributed to the enhanced urinary 11-dehydro-TXB_2_ levels.

The present study found that aspirin 100 mg/d incompletely acetylated COX-1 proteins in CRC tissues, leading to a failure to reduce TXB_2_ levels significantly. These effects were associated with the upregulation of the genes of COX-1 and COX-2 proteins and the EMT marker genes *VIM* and *TWIST1* in colorectal tumor tissue *vs.* predrug (baseline) (Figures 8A, 9A; Supplementary Figure S4A). These are unfavorable responses in patients treated with aspirin since these genes contribute to tumorigenesis, metastasis, invasion, and therapeutic resistance of various tumors (Wang and Dubois, 2010; Brabletz et al., 2018). Differently, aspirin 100 mg/BID or 300 mg/d significantly reduced TXB_2_ levels in the colorectal tumor tissues associated with acetylation of COX-1 comparable to that found in normal tissue; these effects were associated with a restraining impact on the expression of protumorigenic genes (Figures 8B, C, 9B, C; Supplementary Figures S4B, C).

TXA_2_ receptors (TP) are expressed in colorectal tissue, and their activation by TXA_2_ exerts pro-proinflammatory and tumorigenic actions. It was reported that the knocked-down of TBXA2R (TP receptor) or TBXAS1 (TXA_2_ synthase) in human CRC cells led to fewer colonies formation in soft agar than in control cells (Li et al., 2015). We have previously found that TP activation leads to the induction of COX-2, EMT genes, proliferation, and migration of intestinal myofibroblasts (Bruno et al., 2022; Sacco et al., 2019; D’Agostino et al., 2021). The activated fibroblasts promote cancer cell progression and immune evasion through the secretion of immunomodulatory molecules, physical interaction with immune cells, and remodeling of the extracellular matrix (Caligiuri and Tuveson, 2023).

Our finding of increased levels of PGE_2_ in CRC tissue, along with upregulation of the COX-2 gene and downregulation of the 15-PGDH gene, are likely contributing factors to the enhanced urinary PGEM found in CRC (Wang and Dubois, 2013). We showed that all aspirin doses caused variable and marginal acetylation of COX-2 at Serine 516 in CRC tissue. This can explain aspirin’s inability to inhibit the biosynthesis of PGE_2_ in both CRC tissues and *in vivo* (urinary PGEM). However, we assessed % AceCOX-2 at 12–24 h after aspirin dosing. It is possible that a rapid *de novo* protein synthesis of COX-2 in nucleated cells occurred (Patrignani et al., 2017). This event can prevent the detection of a persistent inhibitory effect of COX-2 by aspirin. In contrast, the slow COX-1 turnover in normal nucleated cells of the colorectum (Patrignani et al., 2017) can explain the finding of persistent detection of AceCOX-1 throughout aspirin dosing intervals (12–24 h). We observed that administering aspirin 100 mg/d, but not BID, leads to a lower extent of COX-1 acetylation in cancer tissue than in normal tissue. This may suggest that cancer cells have a faster turnover of COX-1 than normal cells.

Aspirin has a short pharmacokinetics half-life of approximately 20 min (Pedersen and FitzGerald, 1984; Brunton et al., 2017). It is converted into its primary metabolite, SA, a weak prostanoid inhibitor (Joharatnam-Hogan et al., 2023). It has been reported that millimolar concentrations of SA can disrupt multiple cancer-promoting signaling pathways (Dovizio et al., 2013). We found that ASA plasma levels were undetectable 24 h after the last dose of aspirin 100 mg or 300 mg, or 12 h after aspirin 100 mg/BID. In contrast, a few micromolar SA concentrations were measured in plasma (Supplementary Figure S5). We have previously shown that the Cmax of plasma SA concentration after chronic dosing with aspirin 100 mg/day to healthy subjects averages 39 ± 17 μM (Patrignani et al., 2014); it can be increased by 2-3 fold after dosing with aspirin 300 mg/d or 100 mg/BID, considering an elimination half-life of SA between 3 and 12 h at therapeutic doses (Brunton et al., 2017). Based on these data, it seems that the aspirin doses utilized in the current study cannot achieve millimolar levels of plasma SA. Thus, the effects of Aspirin reported in the present study support the role of ASA and not SA.

This study’s limitation is the low percentage of females in the three aspirin groups due to challenges in enrolling patients with CRC during the COVID-19 pandemic and because CRC is more frequent in males. Furthermore, the present study did not explore the potential influence of genetic alterations on changes in biomarkers resulting from different aspirin doses. However, Frouws et al. (2017) have reported that *BRAF* and *KRAS* mutation status cannot be used as a marker for individualized treatment with aspirin. In contrast, it was previously shown that tumors with mutations of *PIK3CA* (protein name: phosphatidylinositol 4,5-bisphosphate 3-kinase catalytic subunit alpha isoform) are more sensitive to the benefits of aspirin (Liao et al., 2012). However, what caused this relationship is yet to be determined.

The study’s strength lies in the deep phenotyping of patients using innovative biomarkers for aspirin’s action. For the first time, the extent of acetylation of COX-1 and COX-2 by aspirin was assessed in CRC patients, and these measurements allowed for the determination of the distribution of aspirin at different doses in platelets *vs.* the colorectum. These assessments showed the direct effects of aspirin on the colorectum. We showed that the inhibitory effect on COX-2 was incomplete, variable, and transient at any dose. Our findings support the contribution of colorectal cancer COX-1 inhibition in aspirin anticancer effects. Chulada et al. (2000) reported that homologous disruption of either *Ptgs1* or *Ptgs2* reduced polyp formation in *Apc* ^*Min/+*^ mice by approximately 80%. In these results, the contribution of platelet COX-1 played a role, as it was demonstrated by Bruno et al. (2022) using *Apc* ^*Min/+*^ mice with selective deletion of COX-1 in platelets. It can be suggested an enhanced anticancer efficacy by nonselective NSAIDs such as Naproxen, which is a potent platelet COX-1 inhibitor (Capone et al., 2004), but can also affect PGE_2_ in colorectal mucosa (Reyes-Uribe et al., 2021) and the systemic biosynthesis of COX-2-dependent prostanoids (Capone et al., 2004). However, the profound impact of Naproxen on normal epithelial COX-1-dependent PGE_2_ biosynthesis, a cytoprotective pathway for the gastrointestinal system, translates into an enhanced risk of upper gastrointestinal complications (Bhala et al., 2013). Thus, using nonselective NSAIDs as an anticancer tool is not recommended.

Our study is innovative, and the results have important clinical implications. Through analyzing the novel, direct biomarkers of drug action, we have discovered the potential to predict how patients will respond to aspirin doses in the context of CRC. This involves assessing the extent of COX-isozyme acetylation in circulating cells and colorectal tissues (Patrignani et al., 2014; Patrignani et al., 2017; Tacconelli et al., 2020). Our findings show that all three doses of aspirin have similar effects in acetylating platelet COX-1 and preventing platelets from contributing to the systemic biosynthesis of TXA_2_ in CRC. However, taking 300 mg/d or 100 mg/BID is more effective than taking 100 mg/d in acetylating COX-1, reducing TXB_2_ levels, and restraining tumor-promoting gene expression in CRC tissue. These results suggest that a higher dose or more frequent daily dosing can improve the anticancer efficacy of 100 mg of aspirin daily. However, further studies are necessary to confirm and validate these findings.

## Data Availability

The raw data supporting the conclusion of this article will be made available by the authors, without undue reservation.
