# Supplementary material for: Optimizing aspirin dose for colorectal cancer patients through deep phenotyping using novel biomarkers of drug action
Source: Front Pharmacol. 2024 Feb 29;15:1362217. doi: 10.3389/fphar.2024.1362217 (PMC10941341; doi:10.3389/fphar.2024.1362217)
Supplement: Supplementary file 1 [file Table1.pdf]

frontiers

Supplementary Material

A

B

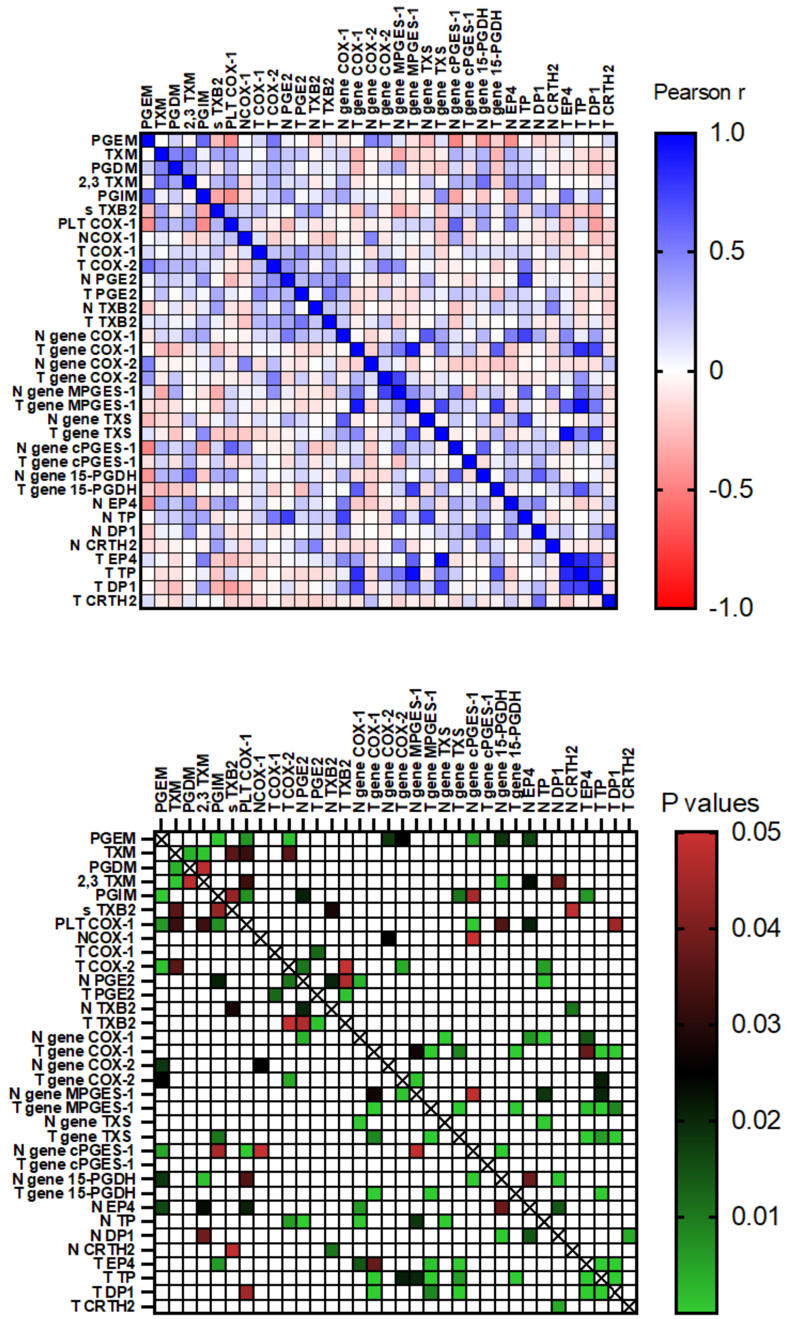

Supplementary Figure S1. Linear regression of the biomarkers analyzed. Heat map of the linear regression Pearson r values (A) and P-values (B) among the urinary levels of prostanoid metabolites, COX-isozyme expression in platelets and normal and tumor colorectal tissue (proteins and mRNAs), prostanoid levels and gene expression of downstream synthases and prostanoid receptors detected at baseline in the 34 CRC patients. Abbreviations: TXM, 11-dehydro-TXB_2; 2,3 TXM, 2,3-dinor-TXB_2; $sTXB_2$, serum TXB_2; PLT COX-1, platelet COX-1; N, normal; T, tumor.

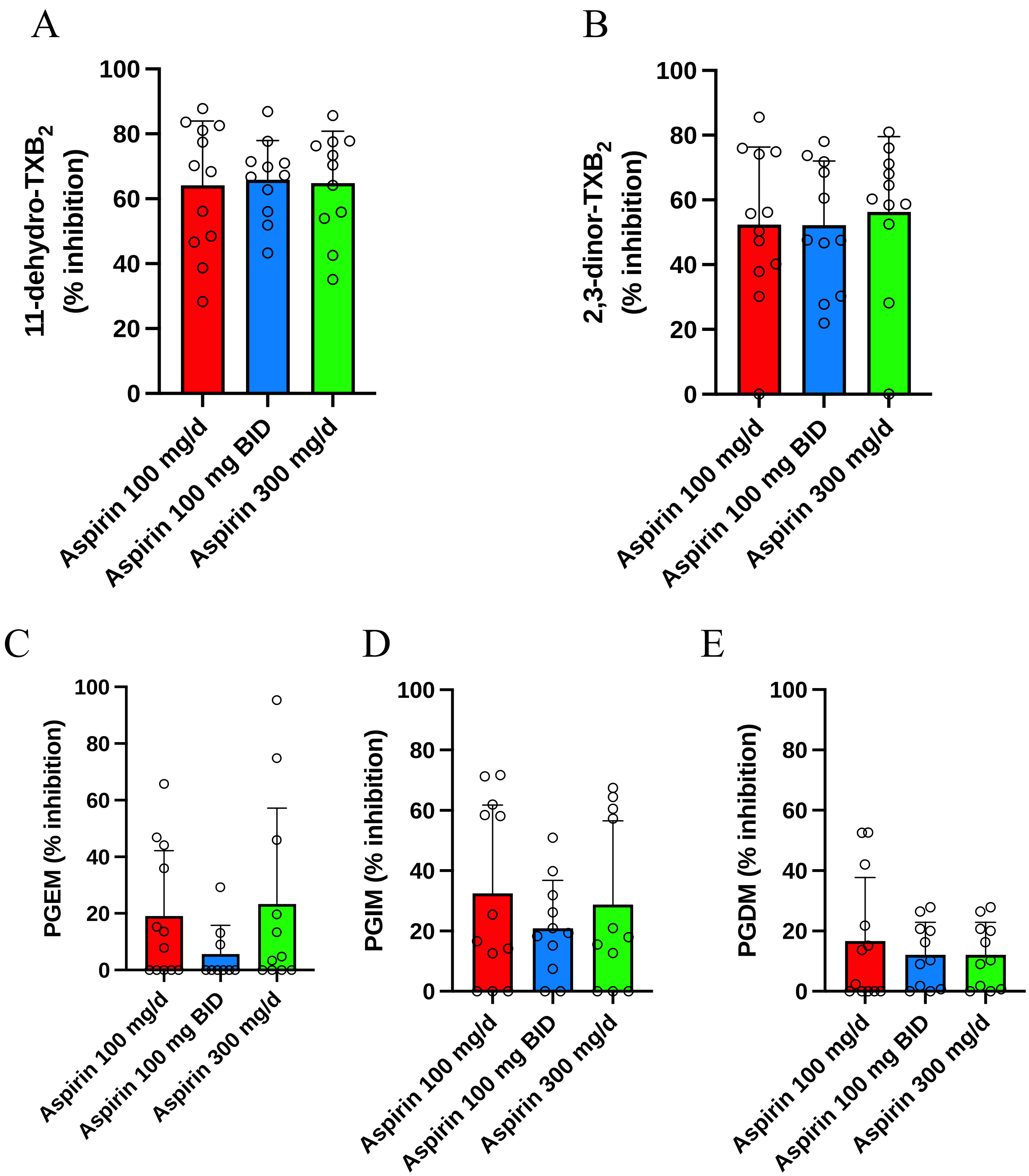

Supplementary Figure S2. Inhibitory effects of different doses of Aspirin (100 mg/d, 100 mg BID, and 300 mg/d) on the systemic biosynthesis of prostanoids in CRC patients. Effects of different doses of Aspirin on the systemic biosynthesis of TXA_2, PGE_2, PGI_2, and PGD_2 by assessing their primary urinary enzymatic metabolites [11-dehydro-TXB_2 (A) and 2,3-dinor-TXB_2 (B), PGEM(C), PGIM(D) and PGDM (E)] by LC-MS/MS. The values were reported as % inhibition from baseline values. All values are shown as scatter dot plots with mean+SD (n = 11, 12). Data were analyzed by one-way ANOVA followed by Tukey's multiple comparisons test.

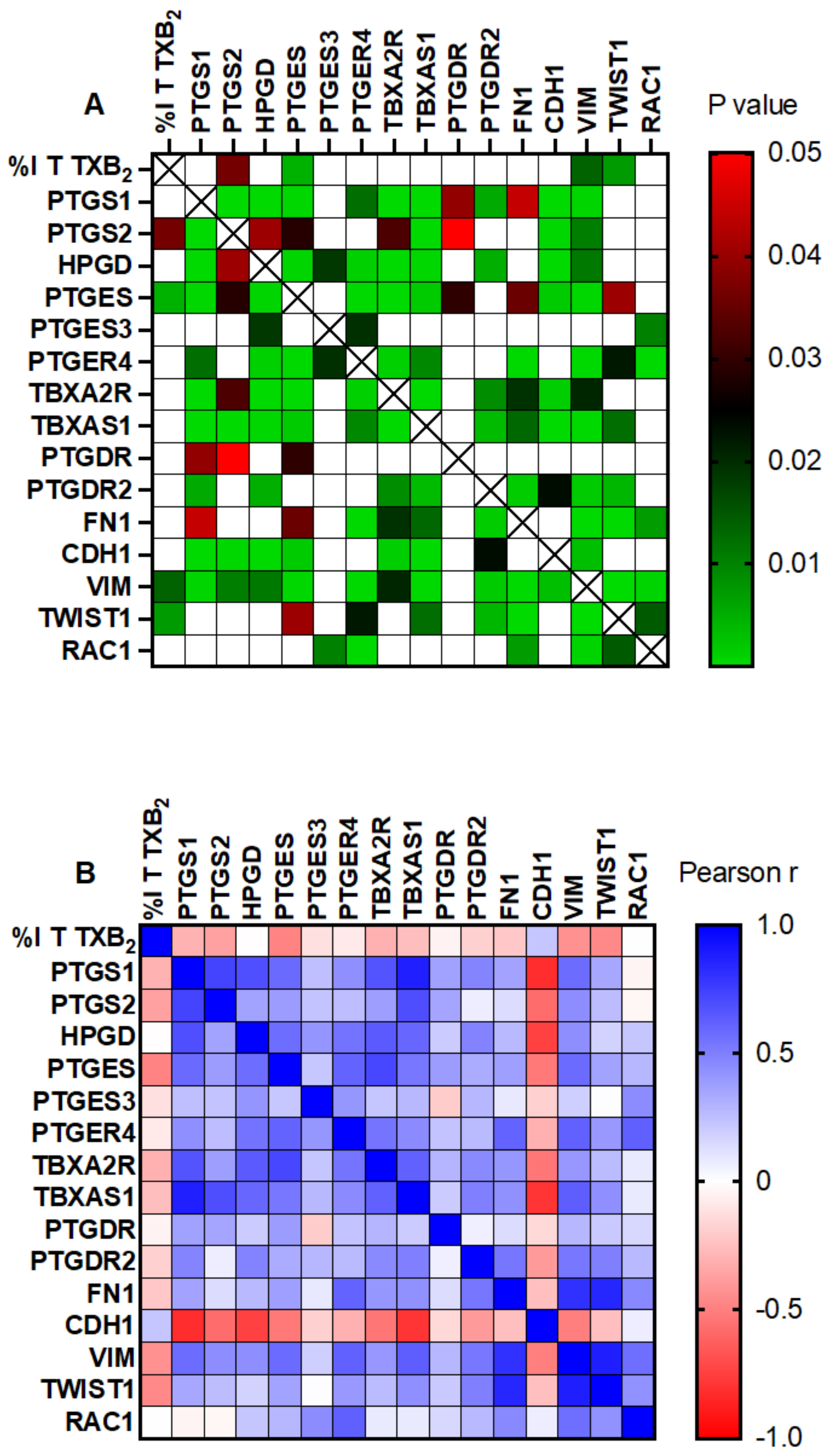

Supplementary Figure S3. Heat map of the linear regression (Pearson) P-values (A) and r values (B) among the % reduction of tumor TXB_2 by Aspirin (all doses) and log_2 fold changes of the expression of the15 genes analyzed in colorectal tumor tissue of the CRC patients treated with Aspirin vs. baseline.

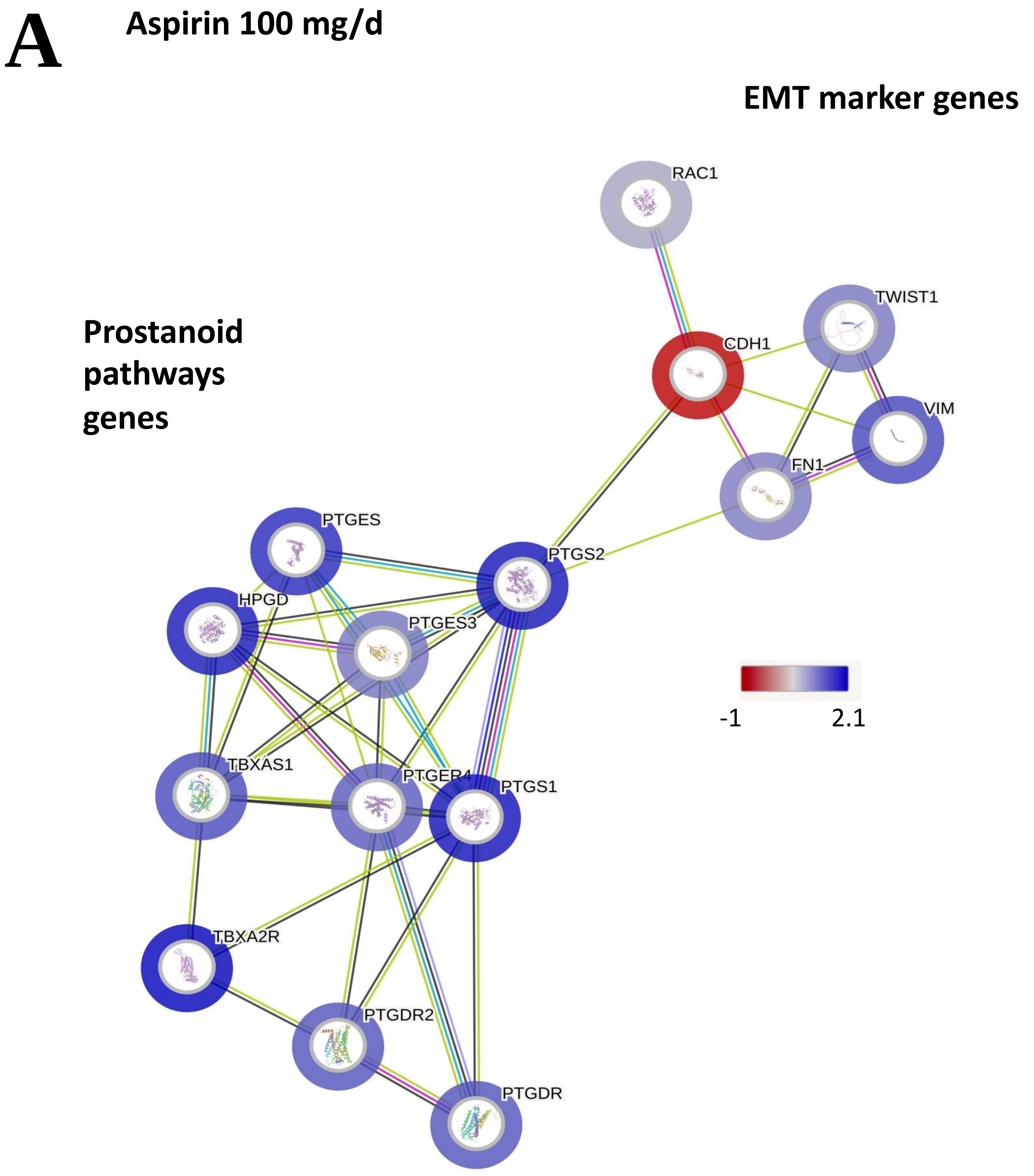
A
Aspirin 100 mg/d
EMT marker genes
Prostanoid pathways genes
RAC1
TWIST1
CDH1
VIM
FN1
PTGES
PTGS2
HPGD
PTGES3
TBXAS1
PTGER4
PTGS1
TBXA2R
PTGDR2
PTGDR
-1
2.1

Figure S4

B

Aspirin 100 mg BID

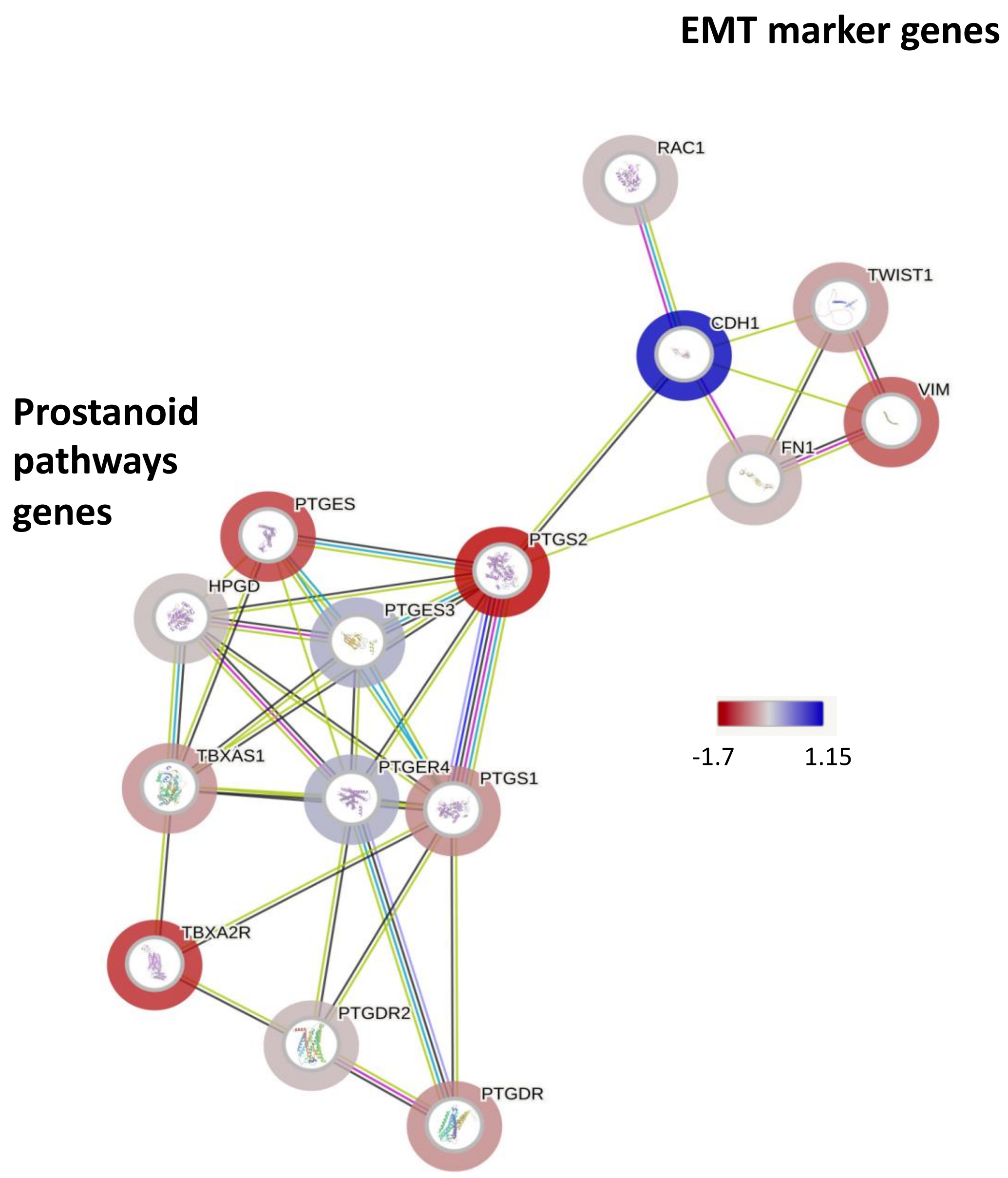

Figure S4

C **Aspirin 300 mg/d**

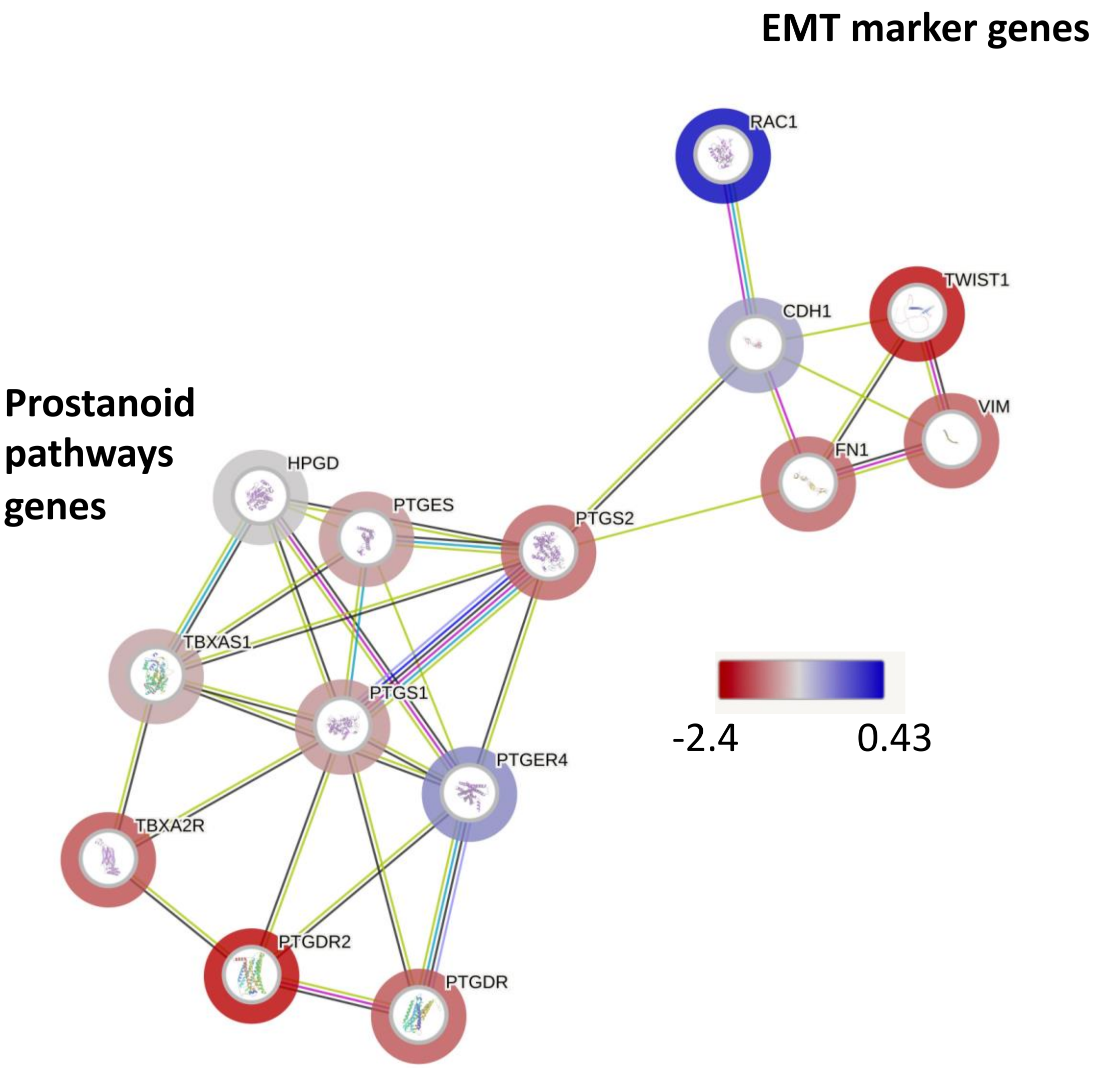

Supplementary Figure S4. The log$_2$(fold change) of the expression of 15 genes assessed in colorectal tumor tissues of CRC patients treated with Aspirin 100 mg/d (**A**), 100 mg/BID (**B**), or 300 mg/d (**C**) *vs.* the values assessed at predrug (baseline) analyzed using STRING v11 for networks. Network nodes represent the proteins produced by a single, protein-coding gene locus. Edges represent protein-protein associations. The halo color is based on the values submitted.

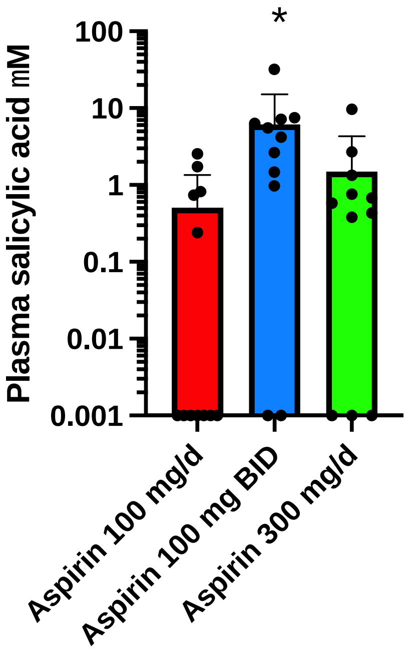

Supplementary Figure S5. Acetylsalicylic acid (ASA) and salicylic acid(SA) assessed in plasma after dosing with Aspirin 100 mg/d, 100 mg BID and 300 mg/d. The levels were assessed by LC-MS/MS, as previously reported [9], at 24 after dosing with 100 or 300 mg/d and 12 h after the last dose of 100 mg BID. ASA levels were undetectable (not shown). All plasma SA values are shown as scatter dot plots with mean+SD (n = 11, 12). Data were analyzed by one-way ANOVA followed by Tukey's multiple comparisons test. *P<0.05 *vs*. 100 mg/d.

Supplementary Table S1. Demographic and baseline clinical features of CRC patients treated with Aspirin 100 mg/d, 100 mg BID, or 300 mg/d

	**ASA, 100mg/d, n=12**	**ASA, 100mg/BID, n=11**	**ASA, 300mg/d, n=11**
**Age, y**	63.00±9.92	68.45±7.98	62.73±10.05
**Sex, % female**	25.00	36.36	9.1
**BMI, kg/m^2**	26.63±3.35	28.64±5.47	28.63±5.69
**Systolic blood pressure, mmHg**	130.8±11.48	125.5±14.71	130.70±13.64
**Diastolic blood pressure, mmHg**	78.75±11.72	78.18±12.00	81.55±8.80
**Leukocytes (x10^3/mm^3)**	7.50±1.76	6.89±2.87	7.79±2.23
**Erythrocytes (x10^6/mm^3)**	4.86±0.31	4.95±0.38	5.15±0.45
**Platelets count (x10^3/mm^3)**	230.8±57.11	237.90±84.44	260.4±72.50
**Mean corpuscular hemoglobin concentration, g/dl**	33.32±0.77	33.48±0.77	33.38±0.73
**Hematocrit, %**	42.88±2.88	43.17±3.81	44.35±5.29
**Haemoglobin, g/dl**	14.28±1.06	14.45±1.27	14.83±1.95
**Aspartate transaminase (AST), IU/l**	20.08±6.8	23.27±5.19	20.73±8.76
**Alanine aminotransferase (ALT), IU/l**	14.00±4.82	17.55±7.43	22.09±10.24 *
**AST/ALT ratio**	1.609±0.74	1.44±0.48	0.99±0.31*
**Gamma-glutamyltransferase, IU/liter**	18.75±10.39	22.27±10.68	37.36±21.77*
**Alkaline phosphatase, IU/l**	70.83±18.21	84.55±24.30	80.55±26.73
**Total Bilirubin, mg/dl**	0.67±0.25	0.81±0.54	0.73±0.23
**Glycemia, mg/dl**	97.08±25.04	116.90±47.55	103.2±10.77
**Creatinine, mg/dl**	0.98±0.18	0.97±0.16	1.00±0.36
**Fibrinogen, mg/dl**	499±82.49	489.10±110.7	563.2±101.4
**Urea, g/l**	0.29±0.09	0.36±0.085	0.33±0.14
**HDL cholesterol, mg/dl**	58.50±19.03	53.27±10.36	47.18±13.65
**LDL cholesterol, mg/dl**	117.2±29.91	108.60±42	125.5±40.04
**Triglycerides, mg/dl**	87.17±24.00	83.09±31	111.80±38.09
Comorbidity			
**Diabetes, n(%)**	2(16.66)	4(36.36)	3(27.27)
**Dyslipidemias, n(%)**	5(41.67)	4(36.36)	5(45.45)
**Hypertension, n(%)**	1(8.33) **	8(72.72)	5(45.45)
Concomitant drugs			
**Statin**	3(25.00)	5(45.45)	5(45.45)
**ACE-inhibitors**	1(8.33)	3(27.27)	1(9.09)
**Sartans**	0(0)	4(36.36)	2(18.18)
**Hypoglycemic**	2(16.66)	4(36.36)	3(27.27)
**Diuretics**	1(8.33)	4(36.36)	2(18.18)
**Beta-adrenergic blocking agents**	0(0)	1(9.09)	0(0)
**Calcium channel blockers**	0(0)	2(18.18)	0(0)
**Alpha-adrenergic blocking agents**	0(0)	3(27.27)	2(18.18)

Values are reported as mean±SD. One-way ANOVA followed by Tukey's post hoc test was used to compare means differences among the 3 groups. The differences in the distribution of categorical variables were assessed by the chi-square test. *P<0.05 *vs.* Aspirin 100 mg/d; **P<0.01 *vs.* Aspirin 100 mg BID.

Supplementary Table S2. TNM staging of colorectal tumors of the CRC patients treated with Aspirin 100 mg/d, 100 mg BID or 300 mg/d

	**Aspirin 100mg/d, n=12**	**Aspirin 100mg BID n=11**	**Aspirin 300mg/d, n=11**
TNM classification			
**T1, n(%)**	0(0)	0(0)	0(0)
**T2, n(%)**	3(25)	1(9.09)	1(9.09)
**T3, n(%)**	9(75)	10(90.9)	7(63.6)
**T4, n(%)**	0(0)	0(0)	3(27.2)
**N0, n(%)**	3(25)	4(36.3)	6(54.5)
**N1, n(%)**	5(41.6)	2(18.1)	1(9.09)
**N2, n(%)**	4(33.3)	5(45.4)	4(36.3)
**N3, n(%)**			
**M0, n(%)**	12(100)	7(63.6)	9(81.8)
**M1, n(%)**	0(0)	4(36.3)	1(9.09)
**Mx, n(%)**	0(0)	0(0)	1(9.09)
Location			
**Cecum**	0(0)	1(9.09)	1(9.09)
**Ascending colon**	0(0)	0(0)	1(9.09)
**Transverse colon**	0(0)	1(9.09)	2(18.1)
**Descending colon**	0(0)	1(9.09)	0(0)
**Sigma**	4(33.3)	1(9.09)	1(9.09)
**Rectum**	5(41.6)	6(54.5)	5(45.4)
**Rectosigmoid junction**	3(25)	1(9.09)	1(9.09)

Supplementary Table S3. Immunohistochemistry staining for the mismatch repair proteins (MMR) in biopsies of CRC patients treated with Aspirin 100 mg/d, 100 mg BID, or 300 mg/d

	**Aspirin 100 mg/d§**	**Aspirin 100 mg BID°**	**Aspirin 300mg/d+**
Mismatch repair proteins (Loss of expression)			
**MLH1, n**	0	1	0
**MSH2, n**	0	0	0
**MSH6, n**	0	0	0
**PMS2, n**	0	1	0

§performed in n=11 patients; °performed in n=7 patients; +performed in n=11 patients.
Values are reported as the number of patients (n).

Supplementary Table S4. Multiple regression associations of urinary 11-dehydro-TXB_2 (TXM) (A), PGEM (B) with other biomarkers and clinical laboratory data

A

Variable	Regression coefficient	95% Confidence Interval (CI)	P value
PLT COX-1	23.15	0.633 to 45.66	0.0443
(T) COX-2	357.00	63.08 to 650.90	0.0191
PGDM	174.90	39.31 to 310.60	0.0136
MCHC	-453.70	-786.90 to -120.60	0.0093

B

Variable	Regression coefficient	95% Confidence Interval (CI)	P value
PGIM	0.01756	0.00823 to 0.02689	0.0007
PLT COX-1	-0.31460	-0.5556 to -0.07371	0.0127
(T) COX-2	7.07100	4.1600 to 9.9820	<0.0001
(N) PGE_2	-0.88950	-1.2950 to -0.4840	0.0001
(T) gene cPGES-1	-1.3760	-2.4410 to -0.3109	0.0136
(N) gene 15-PGDH	-1.1520	-2.1590 to 0.1448	0.0268

Abbreviations: Platelet (PLT), mean corpuscular hemoglobin concentration (MCHC), normal (N), tumor (T) colorectal tissue

Supplementary Table S5. Multiple regression associations of urinary PGDM (A), PGIM (B) with other biomarkers and clinical laboratory data

A

Variable	Regression coefficient	95% Confidence Interval (CI)	P value
TXM	6.667	1.26 to 12.21	0.0254
SBP	0.0009635	0.0001277 to 0.001799	0.0430
DBP	-0.04629	-0.09101 to -0.001565	0.0309
Glu	0.05885	0.005824 to 0.1119	0.0252
LDL	-0.01647	-0.03073 to 0.002199	0.0370

B

Variable	Regression coefficient	95% Confidence Interval (CI)	P value
PGEM	13.94	6.23-21-65	0.0009

Abbreviations: Glucose (Glu), systolic blood pressure (SBP), diastolic blood pressure (DBP), low-density lipoprotein (LDL)
